# Tumor-Stroma-Inflammation Networks Promote Pro-metastatic Chemokines and Aggressiveness Characteristics in Triple-Negative Breast Cancer

**DOI:** 10.3389/fimmu.2019.00757

**Published:** 2019-04-12

**Authors:** Yulia Liubomirski, Shalom Lerrer, Tsipi Meshel, Linor Rubinstein-Achiasaf, Dina Morein, Stefan Wiemann, Cindy Körner, Adit Ben-Baruch

**Affiliations:** ^1^School of Molecular Cell Biology and Biotechnology, George S. Wise Faculty of Life Sciences, Tel Aviv University, Tel Aviv, Israel; ^2^Division of Molecular Genome Analysis, German Cancer Research Center, Heidelberg, Germany

**Keywords:** cancer-associated fibroblasts, CCL2, CCL5, CXCL8, interleukin 1β, mesenchymal stem cells, triple-negative breast cancer, tumor necrosis factor α

## Abstract

The tumor microenvironment (TME) plays key roles in promoting disease progression in the aggressive triple-negative subtype of breast cancer (TNBC; Basal/Basal-like). Here, we took an integrative approach and determined the impact of tumor-stroma-inflammation networks on pro-metastatic phenotypes in TNBC. With the TCGA dataset we found that the pro-inflammatory cytokines tumor necrosis factor α (TNFα) and interleukin 1β (IL-1β), as well as their target pro-metastatic chemokines CXCL8 (IL-8), CCL2 (MCP-1), and CCL5 (RANTES) were expressed at significantly higher levels in basal patients than luminal-A patients. Then, we found that TNFα- or IL-1β-stimulated co-cultures of TNBC cells (MDA-MB-231, MDA-MB-468, BT-549) with mesenchymal stem cells (MSCs) expressed significantly higher levels of CXCL8 compared to non-stimulated co-cultures or each cell type alone, with or without cytokine stimulation. CXCL8 was also up-regulated in TNBC co-cultures with breast cancer-associated fibroblasts (CAFs) derived from patients. CCL2 and CCL5 also reached the highest expression levels in TNFα/IL-1β-stimulated TNBC:MSC/CAF co-cultures. The elevations in CXCL8 and CCL2 expression partly depended on direct physical contacts between the tumor cells and the MSCs/CAFs, whereas CCL5 up-regulation was entirely dependent on cell-to-cell contacts. Supernatants of TNFα-stimulated TNBC:MSC “Contact” co-cultures induced robust endothelial cell migration and sprouting. TNBC cells co-cultured with MSCs and TNFα gained migration-related morphology and potent migratory properties; they also became more invasive when co-cultured with MSCs/CAFs in the presence of TNFα. Using siRNA to CXCL8, we found that CXCL8 was significantly involved in mediating the pro-metastatic activities gained by TNFα-stimulated TNBC:MSC “Contact” co-cultures: angiogenesis, migration-related morphology of the tumor cells, as well as cancer cell migration and invasion. Importantly, TNFα stimulation of TNBC:MSC “Contact” co-cultures *in vitro* has increased the aggressiveness of the tumor cells *in vivo*, leading to higher incidence of mice with lung metastases than non-stimulated TNBC:MSC co-cultures. Similar tumor-stromal-inflammation networks established in-culture with luminal-A cells demonstrated less effective or differently-active pro-metastatic functions than those of TNBC cells. Overall, our studies identify novel tumor-stroma-inflammation networks that may promote TNBC aggressiveness by increasing the pro-malignancy potential of the TME and of the tumor cells themselves, and reveal key roles for CXCL8 in mediating these metastasis-promoting activities.

## Introduction

Breast cancer is a common malignant disease classified into several subtypes that differ in their markers, molecular characteristics and prognosis. Tumors of the triple-negative subtype of breast cancer (TNBC; generally corresponding to the “Basal/Basal-like” subtype of patient datasets, determined by PAM50 gene signatures) lack the expression of estrogen receptor α, progesterone receptor and HER2, are highly aggressive and are most likely to recur. Unlike the luminal-A tumors that are characterized by better survival, or the HER2+ tumors, TNBC/basal tumors cannot be treated by receptor-targeted therapies and demonstrate high relapse rates following chemotherapy (1–4).

As with other malignancies, breast tumors develop and progress within an intimate tumor microenvironment (TME) (5–8). Recent studies indicate that stromal cells, including mesenchymal stem cells (MSCs) and cancer-associated fibroblasts (CAFs) are key regulators of tumor progression in cancer (9, 10). In general, MSCs enrich the TME with tumor-promoting factors, and endow the tumor cells with improved abilities to invade and generate metastases; MSCs also undergo transition to CAFs that promote breast cancer/TNBC progression (11–18). Particularly in TNBC, MSCs contribute to higher aggressiveness by promoting the expression of angiogenic factors and pro-metastatic chemokines such as the pro-inflammatory chemokines CXCL8, CCL2 and CCL5 (or their murine counterparts) (19–26). The axes established by these chemokines and their receptors are well-known for their pro-tumorigenic roles, including in TNBC [e.g., (26–41)].

Tumor-educated MSCs evolve within a TME enriched with inflammatory cells and pro-inflammatory cytokines, which generally promote breast cancer progression (42–44). Two such pro-inflammatory cytokines are tumor necrosis factor α (TNFα) and interleukin 1β (IL-1β). It was demonstrated that despite its potential anti-tumor cytotoxic activities, chronic presence of TNFα in tumors has led to tumor progression. Joined with the pro-angiogenic and pro-inflammatory activities of IL-1β, the two cytokines were identified as pro-metastatic factors in many tumor types (45–49). In TNBC, sequencing, serum-profiling and immunohistochemistry studies of small patient cohorts provided initial evidence to relevance of high expression of the two cytokines or of their signaling components to metastasis in TNBC patients (50–53); in parallel, studies in animal models have demonstrated causative tumor-promoting roles for TNFα and IL-1β in TNBC (52, 54–59). TNFα and IL-1β were also connected with pro-malignancy activities of MSCs and CAFs in TNBC (21, 60–65). Specifically with regards to chemokines, we and others have shown that TNFα and IL-1β elevated the expression of CXCL8, CCL2 and CCL5 (or their murine counterparts) by MSCs [(65–69); the degree of increase depended on assay conditions], thus promoting the pro-inflammatory and pro-malignancy phenotype of these stromal cells.

However, to date, we still lack understanding of the interactions that are established between TNBC cells, stromal cells and their intimate pro-inflammatory TME, and we do not have enough information on the way such interactions affect disease course. Specifically, it is not known whether the pro-metastatic characteristics of the TME and of the tumor cells are increased when TNBC cells interact with MSCs/CAFs in the presence of pro-inflammatory stimuli. Thus, the aim of our present study was to identify the influence of the tumor-stroma-inflammation triage on the content of pro-metastatic chemokines as proxies for potential pro-malignancy activities that enrich the TME of TNBC tumors, as well as on angiogenesis and on the migratory, invasive and metastatic properties of TNBC cells.

In view of their high relevance to tumor progression in TNBC, TNFα and IL-1β were selected as representatives of the pro-inflammatory TME in our study. Here, we demonstrate that stimulation of TNBC:stroma co-cultures by these two cytokines has led to increased pro-metastatic activities at multiple levels, including: expression levels of the chemokines CXCL8, CCL2 and CCL5, angiogenesis, cancer cell morphology, tumor cell migration and tumor cell invasion. Importantly, we found that CXCL8 was a key regulator of the pro-metastatic activities that came into play in the TNBC-stroma-inflammation networks, including angiogenesis, metastasis-related morphology, tumor cell migration and invasion of TNBC cells. Moreover, the tumor-stroma-inflammation network has promoted the metastatic potential of TNBC cells and has led to elevated metastasis *in vivo*. Parallel in-culture studies that were performed with tumor-stroma-inflammation networks established with luminal-A cells demonstrated that in general they were less potent or differently active than those established with TNBC cells.

Thus, our findings set the pro-inflammatory inputs acting at the tumor:stroma interface, and their pro-metastatic outputs, as targets for improved therapy in TNBC. Since inhibitors of TNFα and IL-1β are used vastly in the treatment of inflammatory diseases (70–72), our findings suggest that these two cytokines may be considered as novel therapeutic modalities in TNBC. Such an approach, combined with the use of chemokine receptor inhibitors [e.g., of the CXCL8 receptors CXCR1 or CXCR2; (39, 73)], may prevent tumor-stroma interactions that increase metastasis in TNBC.

## Materials and Methods

### Analyses of Patient Datasets

RNAseq-based gene expression analyses of breast cancer patient data were performed with the TCGA dataset (74). Subtypes were defined based on the PAM50 annotation file provided within the dataset: Basal (often overlapping the term TNBC): 141 patients; Luminal-A: 421 patients. Gene expression levels of TNFα, IL-1β, CXCL8, CCL2 and CCL5 were determined. In all analyses, log2-transformed expression values of the genes were presented. Statistical analyses were performed following Shapiro-Wilk test, determining the normality of distribution for each gene by individual subtype. Comparisons of gene expression levels between the two clinical subtypes were presented in boxplots. *p*-values were determined by two-tailed Mann-Whitney test. The distribution of gene expression levels in basal and luminal-A patients was presented in histograms, where statistical analyses were performed by two-tailed Mann-Whitney test. In studies correlating the expression levels of the different genes, correlation coefficients and *p*-values were analyzed using Spearman correlation. Values of *p* ≤ 0.05 were considered statistically significant.

### Breast Tumor Cell Lines and Stromal Cells

The human TNBC cell lines (all from ATCC) included: MDA-MB-231 and MDA-MB-468 cells that were grown in DMEM (Gibco, Life technologies, Grand island, NY); BT-549 cells that were grown in RPMI 1640 medium (Biological Industries, Beit Ha'emek, Israel). Media were supplemented with 10% fetal bovine serum (FBS) and 1% penicillin-streptomycin solution (Biological Industries); for BT-549 cells, recombinant human (rh) insulin (10 mg/ml; #I9278; Sigma-Aldrich, Merck KGaA, Darmstadt, Germany) was added to the medium. The human luminal-A cell lines MCF-7 (from ATCC) and T47D [provided by Dr. Keydar who generated the cell line (75)] were grown in culture in the same medium as MDA-MB-231 cells. Human pulmonary microvascular endothelial ST1.6R cells (HPMEC) were kindly provided by Dr. Unger and Dr. Kirkpatrick, Institute of Pathology, Johannes-Gutenberg University, Mainz, Germany. These cells were grown as described in Krump-Konvalinkova et al. (76), with minor modifications.

Human bone marrow-derived MSCs were purchased from Lonza (#PT-2501; Walkersville, MD), which validated them as MSCs based on cell markers and differentiation potential. Routine growth of MSCs took place in mesenchymal stem cell growth medium (#PT-3001; Lonza) or in MesenCult (#05411; Stemcell Technologies Inc., Vancouver, BC, Canada) and they were used for up to 10 passages. In this study, MSCs of four different healthy donors were used. Patient-derived CAFs from a primary breast tumor (used in ELISA and their accompanying signaling experiments) and from a lung metastasis (used in tumor cell invasion assays) were kindly provided by Dr. Bar, Sheba Medical Center, Ramat Gan, Israel). The cells were grown, identified and immortalized as described in Katanov et al. (67).

### TNFα and IL-1β Concentrations Used in Different Analyses

Titration studies were initiated by determining the ability of rhTNFα (#300-01A, PeproTech, Rocky Hill, NJ), and rhIL-1β (#200-01B, PeproTech) to elevate in MDA-MB-231 cells and/or MSCs/CAFs the expression of CXCL8, CCL2 and/or CCL5 to levels that enabled us to perform the required comparisons between different cell combinations in ELISA studies (concentrations studied - TNFα: 100 pg/ml, 1 ng/ml, 10 ng/ml; IL-1β: 20, 100, 250, 350, 500, 750 pg/ml). The selected concentrations of 10 ng/ml TNFα and 350 pg/ml IL-1β were appropriate also for MSC and CAF experiments. Therefore, in all MDA-MB-231 studies, alone or with MSC/CAF, these selected concentrations were used in *in vitro* and *in vivo* experiments.

In parallel, titration studies indicated that the above selected concentrations were not optimal for ELISA responses of BT-549 and MDA-MB-468 cells; thus, based on additional analyses, the concentrations of cytokines were raised in these two cell types: MDA-MB-468 cells - 50 ng/ml TNFα and 500 pg/ml IL-1β; BT-549 cells - 25 ng/ml TNFα and 350 pg/ml IL-1β. These selected cytokine concentrations were used in all studies of MDA-MB-468 and BT-549 cells, alone or with MSCs.

The effects of TNFα and IL-1β on morphological changes, angiogenesis, migration and invasion with MCF-7 cells were determined in the same concentrations as used for MDA-MB-231 cells (10 ng/ml TNFα and 350 pg/ml IL-1β). In ELISA studies (and their accompanying signaling experiments) in MCF-7 and T47D cells cytokine concentrations were raised to 50 ng/ml TNFα and 500 pg/ml IL-1β. Although published data [e.g., (77, 78)] and our past studies indicated that lower TNFα and IL-1β concentrations (as in MDA-MB-231 cells) induce signaling and up-regulate the levels of CXCL8, CCL2 and CCL5 in MCF-7 and T47D cells, we expected that these cytokine concentrations will not enable us to clearly distinguish “intermediate” levels of chemokine induction in ELISA assays. Thus, cytokine concentrations were increased, as described above.

### Western Blot Studies

Based on kinetics analyses (Data not shown), cells were stimulated for 15 min by TNFα or IL-1β (concentrations as described above) or their vehicle (similar for both cytokines), in medium containing 0.5% FBS. The cells were lysed in RIPA lysis buffer, followed by conventional Western blot (WB) procedures. The following antibodies (Abs) were used: Phosphorylated (P)-p65 [#3033; Cell Signaling Technology (CST), Danvers, MA]; Total (T)-p65 (#8242; CST); P-JNK (#4668; CST); T-JNK (#9258; CST). Abs directed against GAPDH (#ab9485; Abcam, Cambridge, UK) served for loading controls. The membranes were reacted with streptavidin-horseradish peroxidase (HRP)-conjugated goat anti-rabbit IgG (#111-035-003; Jackson ImmunoResearch Laboratories, West Grove, PA), and were subjected to enhanced chemiluminescence (#20-500, Biological Industries).

### Cell Stimulation in Co-culture Experiments

TNBC or luminal-A cells were grown in “Contact” conditions with MSCs/CAFs (10:1 ratio) in 6-well plates (#3516, Corning, Kennebunk, ME). When relevant, “Transwell” co-cultures were grown in similar plates, in which the two cell types were separated by an insert of 0.4 μm permeable polycarbonate membrane (#3412, Corning). In parallel, each cell type was grown individually in the same cell numbers as in co-cultures, in similar plates. The cells were grown in medium containing 10% FBS for 12 h, and were then treated by TNFα or IL-1β (concentrations as described above) or their vehicle for 7 h in medium supplemented with 0.5% FBS. Then, media were replaced by cytokine-free media (with 0.5% FBS) for additional 60 h. Cell conditioned media (CM) were removed and taken for ELISA assays. When indicated, CM and cell lysates were produced from cells that were subjected to gene down-regulation by siRNA, as detailed below. Cell lysates were produced from cells grown in larger vessels 7 h or 15 min after the beginning of cytokine stimulation and were used in quantitative real-time PCR analyses (qRT-PCR) or in WB studies.

### ELISA Assays

CM obtained from different stimulatory conditions were cleared by centrifugation, followed by determining the expression levels of CXCL8, CCL2 and CCL5 by ELISA. Standard curves at the linear range of absorbance were produced with rhCXCL8, rhCCL2 and rhCCL5 (#200-8M, #300-04 and #300-06, respectively; PeproTech). The following Abs were used (all from PeproTech, unless otherwise indicated): For CXCL8 - Coating Abs: #500-P28; Detecting Abs: #500-P28Bt. For CCL2 - Coating Abs: #500-M71; Detecting Abs: #500-P34Bt. For CCL5 - Coating Abs: #500-M75; Detecting Abs: #BAF278 (R&D Systems, Inc., Minneapolis, MN). HRP-conjugated Streptavidin (#016-030-084; Jackson Immunoresearch laboratories) was added, followed by the substrate TMB/E solution (#ES001; Millipore, Temecula, CA); then, the reaction was stopped by addition of 0.18 M H_2_SO_4_ and absorbance was measured at 450 nm. To provide data on the contents of chemokines generated in each of the treatments and to clearly denote the differences in chemokine production between the different conditions, the findings are presented in ng/ml.

In reversibility experiments, MDA-MB-231:MSC “Contact” co-cultures were stimulated by 10 ng/ml TNFα for 67 h or exposed to vehicle control. Media were replaced and cell growth was continued for 10–14 days in TNFα-free media. CM that were collected following 67 h of stimulation, and 10–14 days after cytokine removal, were subjected to ELISA analysis of CXCL8 expression as described above.

### qRT-PCR Analyses

Total RNA was extracted using the EZ-RNA kit (#20-400; Biological Industries). Using the M-MLV reverse transcriptase (#AM2044; Ambion, Austin, TX), first-strand cDNA was generated from RNA samples. cDNA targets were quantified by qRT-PCR on Rotor Gene 6000 (Corbett Life Science, Concorde, NSW, Australia). Absolute Blue qPCR SYBR Green ROX mix (#AB-4163/A; Thermo Fisher Scientific, Waltham, MA) was used to detect transcripts, according to manufacturer's instructions. Two pairs of specific primers were used (Supplementary Table 1), designed to span different exons. Data were normalized to the housekeeping gene GAPDH. Dissociation curves for each primer set indicated a single product, and “no-template” controls were negative after the 40 cycles used for analysis. Quantification was performed by standard curves, within the linear range of quantification.

### Endothelial Cell Migration and Sprouting

To generate CM for functional *in vitro* angiogenesis assays, MDA-MB-231:MSC and MCF-7:MSC “Contact” co-cultures (10:1 cell ratio in each) were stimulated by TNFα for 7 h; in parallel, CM were produced from tumor cells that were treated by vehicle, from tumor cells stimulated by TNFα, or from tumor cells grown with MSCs only. Media were removed and the cells were cultured for additional 60 h in TNFα-free medium (with 0.5% FBS). CM were collected, cleared by filtration through a 0.45 μm membrane, and loaded in the lower part of migration transwells (#3422, Corning) with 8-μm pore membranes. The migration of HPMEC cells in direction of the different CM was determined after up to 90 min. At the end of the experiments, cells were removed from the upper side of the membranes, the membranes were fixed in ice-cold methanol and stained by Hemacolor (#1.11661; Merck). Cells that transmigrated to the lower side of the membranes in multiple bright fields were counted.

In parallel, CM were also used in sprouting assays of mCherry-expressing HPMECs out of 3D multicellular spheroids (all generated by the same cell number) that were formed in hanging drops for 24 h (79) and embedded into collagen type I (1.3 mg/ml; #354236; Corning). Sprouting of endothelial cells in response to CM was visualized by fluorescent microscopy after 10 days, and was determined quantitatively in multiple spheroids by mCherry signals of cells that sprouted out of spheroid core, quantified by ImageJ.

### Tumor Cell Morphology, Migration and Invasion

In morphology assays, “Contact” co-cultures of mCherry-expressing tumor cells - MDA-MB-231 or MCF-7 - and MSCs (ratio 10:1) were stimulated by TNFα for 67 h; in parallel, mCherry-tumor cells were grown with vehicle, with TNFα only or with MSCs only. Then, tumor cell morphology was determined by fluorescent microscopy at x100 magnification, in multiple fields. In reversibility experiments of MDA-MB-231 cells, media were replaced in “Contact” co-cultures after 67 h, and cell growth was continued for 10–14 days in cytokine-free medium. Morphology was determined following 67 h of stimulation, and 10–14 days after cytokine removal.

Migration assays of MDA-MB-231 cells were performed in transwells with 8-μm pore membranes (#3422, Corning). In these assays, mCherry-MDA-MB-231 cells and MSCs (ratio 10:1) were added to the upper part of the chambers, in the presence of TNFα (10 ng/ml) in serum-free medium. The same number of mCherry-MDA-MB-231 cells were cultured in parallel transwells, in the presence of vehicle control or of TNFα alone. Migration was performed for 12 h toward medium containing 10% FBS [containing TNFα (10 ng/ml) or vehicle control, as appropriate]. The numbers of migrating tumor cells were determined by staining with rabbit Abs to RPF (recognizing mCherry; #PM005; MBL, Woburn, MA), followed by DyLight 550-conjugated Donkey Abs recognizing rabbit IgG (#ab96920, Abcam). Cells were removed from the upper side of the membranes and fixed in ice-cold methanol. Based on DyLight 550 signals and Hemacolor staining used in preliminary analyses (Data not shown), we validated that close to 100% of migrating cells were the tumor cells. Photos of multiple high power fluorescent fields were taken at × 100 magnification. Cells that transmigrated to the lower side of the membranes in multiple fluorescence fields were counted.

Migration of Hoechst-labeled MCF-7 cells was determined by using the same experimental groups as in TNBC studies. MCF-7 cells are known as having a relatively low basal migratory potential, thus appropriate conditions were set, based on published studies and preliminary analyses in our lab. Thus, the membranes were coated with fibronectin for 1 h (20 μg/ml; #03-090-1, Biological Industries), cells were loaded to the upper part and after 21 h photos were taken. Hoechst-expressing tumor cells that migrated to the lower side of the membranes were counted in multiple fields.

Tumor cell invasion assays were performed using 3D multicellular spheroids that were generated for 72 h in hanging-drops [(79), with minor modifications]. Spheroids of “Contact” co-cultures, consisting of mCherry-MDA-MB-231 cells with MSCs/CAFs (ratio 10:1) were embedded into matrigel (9–10.5 mg/ml; #356234, Corning) and were stimulated by TNFα (10 ng/ml) or vehicle. Spheroids were also formed with mCherry-MDA-MB-231 cells alone, and treated by TNFα or by vehicle (same number of cells as in co-cultures). Invasion of mCherry-MDA-MB-231 cells was determined 48 h after the addition of cytokine stimulation (or vehicle). Multiple spheroids were photographed in fluorescent fields at x40 magnification. The invaded area was determined by the mCherry signals of cells that invaded out of spheroid core, quantified by ImageJ. Invasion of MCF-7 cells was determined in a similar manner after 96 h, using mCherry-MCF-7 cells and patient-derived CAFs, in the presence of TNFα (10 ng/ml) or its vehicle control. Spheroids were photographed in fluorescent fields 96 h after the addition of stimulation, at × 40 magnification.

### CXCL8 Down-Regulation by siRNA

Knock-down of CXCL8 expression by transient siRNA transfections was performed in both MDA-MB-231 cells and MSCs, using the Lipofectamine RNAiMAX transfection reagent (#56531; Invitrogen, Grand Island, NY) according to manufacturer's instructions. ON-TARGET plus siRNA CXCL8 SMART pool and non-targeting control siRNA pool (siCTRL) were used (#L-004756-00 and #D-001810-10, respectively; Dharmacon, Lafayette, CO). The efficacy of CXCL8 down-regulation was validated by qRT-PCR or ELISA [Data not shown; 80–90% as in (80)] and the cells were used in assays, as necessary.

### Tumor Growth and Metastasis

“Contact” co-cultures of mCherry-MDA-MB-231 cells with MSCs (10:1 ratio) were stimulated by TNFα or vehicle control, in 0.5% FBS-containing medium for 67 h. In each group (Group 1: Co-culture with TNFα; Group 2: Co-culture with vehicle), the same amount of live co-cultured cells was mixed 1:1 with matrigel (final concentration 4.5 mg/ml; #356234; Corning). The cells were administered orthotopically to the mammary fat pads of female athymic nude mice (#NUDE242; Envigo RMS, Jerusalem, Israel). During the experiment, tumors of Group 1 were supplemented every 3 days by CM taken from MDA-MB-231:MSC “Contact” co-cultures; these CM were generated as follows: The co-cultures were stimulated for 7 h with TNFα (10 ng/ml), after which the cytokine was removed, and CM were collected after additional 60 h of growth in cytokine-free and serum-free medium. These CM were filtered, concentrated × 10, and administered in proximity to tumors. Cytokine- and serum-free media that were put in parallel flasks have undergone similar procedures, and were administered to tumors of Group 2. At the endpoint of experiments (when tumors reached detectable sizes and had to be removed prior to necrosis), approximately 30 days after tumor cell inoculation, mice were sacrificed and primary tumors and lungs were excised. Tumor weights were determined, and tumor volumes were calculated based on caliper measurements. Lung metastases were determined *ex vivo* by the CRi Maestro non-invasive intravital imaging system. The total number of mice that were included in two biological repeats were 12 in Group 1, and 11 in Group 2. Statistical analyses of primary tumor weight and volume were performed by two-tailed unpaired Student's *t*-test. The proportions of mice bearing lung metastases were compared by Fisher's exact test. Procedures involving experimental animals were approved by Tel Aviv University Ethics Committee, and were performed in compliance with local animal welfare laws, guidelines and policies.

### Data Presentation and Statistical Analyses

The statistical analyses of TCGA analyses and *in vivo* experiments were described in their respective sections. *In vitro* experiments were performed in *n* ≥ 3 independent experimental repeats, with MSCs from ≥2 different donors, as indicated in respective figure legends. The results of ELISA, qRT-PCR, WB, HPMEC sprouting and tumor cell migration and invasion assays were compared by two-tailed unpaired Student's *t*-tests. Values of *p* ≤ 0.05 were considered statistically significant. Adjustment for multiplicity of comparisons was done using the Benjamini-Hochberg procedure controlling the FDR at 0.05. All the significant results remained statistically significant after correcting for their multiplicity, except for some of the WB results. It these latter cases lack of significance was due to high variance between the intensities of effects of the experimental repeats of the test, despite the fact that they all demonstrated the same trend. Thus, in presentation of WB analyses we demonstrate not only the average and standard deviations (SD) of the experimental repeats but also the level of effect in each experiment.

## Results

### High Expression Levels of TNFα and IL-1β Are Noted in Tumors of Basal Patients and Are Significantly Coordinated With High Expression Levels of CXCL8, CCL2 and CCL5

To identify the roles of TNFα and IL-1β in regulating tumor-stroma interactions in TNBC, we have extended currently-available studies on TNFα and IL-1β in TNBC patients (50–52) and compared the expression levels of TNFα and IL-1β in two subtypes of breast tumors: (1) Basal tumors, corresponding to the TNBC subtype, which has a most aggressive phenotype; (2) Luminal-A tumors having the best prognosis of all breast cancer subtypes.

**Figure 1:**
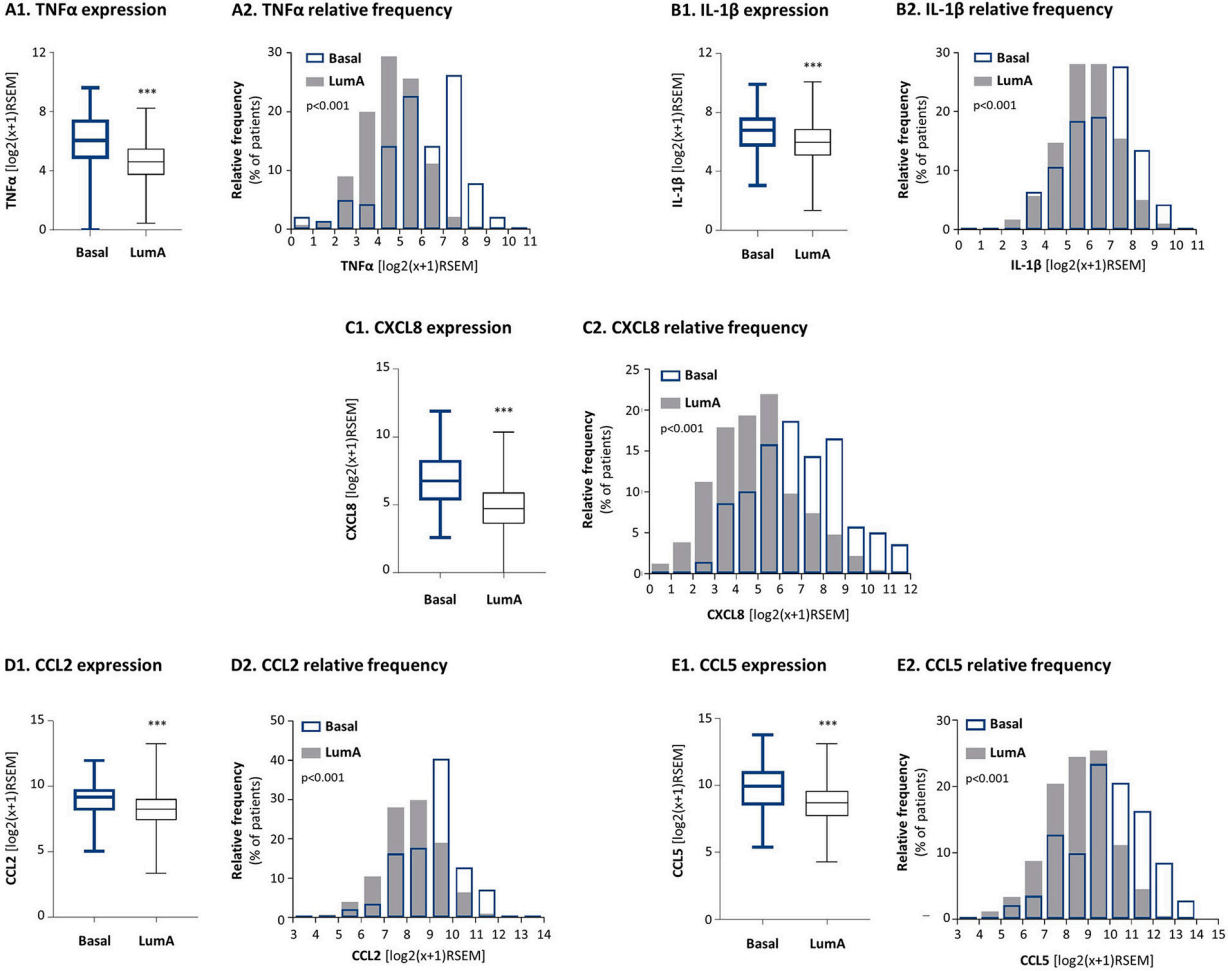
The expression levels of the pro-inflammatory cytokines TNFα and IL-1β and of their chemokine targets are significantly higher in basal patients than in luminal-A patients. The Figure demonstrates gene expression analyses, performed with the TCGA breast cancer dataset. **(A)** TNFα, **(B)** IL-1β, **(C)** CXCL8, **(D)** CCL2, **(E)** CCL5. **(A1–E1)** Boxplots comparing expression levels in basal patients and luminal-A patients. ***p < 0.001. **(A2–E2)** Histograms demonstrating the distribution of expression levels of each of the factors in basal and luminal-A patient tumors. RSEM, RNAseq by expectation-maximization.

Here, by using the TCGA breast cancer dataset we found that TNFα and IL-1β were expressed in significantly higher levels in basal tumors than in luminal-A tumors (Figures 1A1,B1). Distribution analyses (Figures 1A2,B2) demonstrated a larger proportion of basal patients with high TNFα and IL-1β expression levels, than luminal-A patients. In parallel, we analyzed the expression of pro-inflammatory and pro-metastatic chemokines CXCL8, CCL2 and CCL5, chosen as proxies for pro-tumorigenic factors that may be enriched in basal patients due to pro-inflammatory signals. These studies demonstrated significantly higher levels of the three chemokines in basal patients than in luminal-A patients (Figures 1C–E).

**Figure 2:**
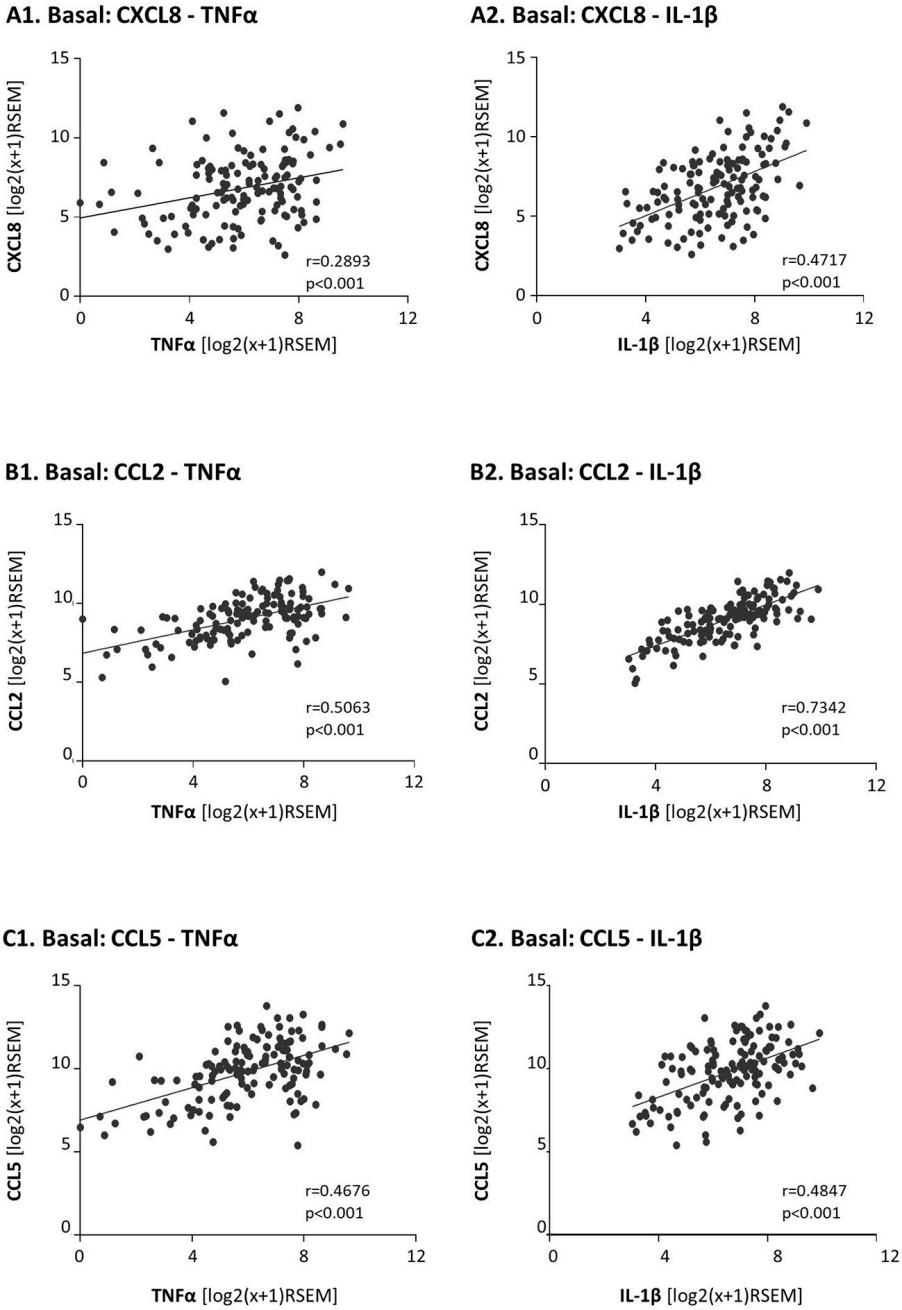
In basal patients, the expression levels of the pro-inflammatory cytokines TNFα and IL-1β are significantly coordinated with the expression levels of their chemokine targets. The Figure demonstrates correlation analyses of gene expression in basal patients, performed with the TCGA breast cancer dataset. **(A)** CXCL8 correlations with TNFα **(A1)** and IL-1β **(A2)**. **(B)** CCL2 correlations with TNFα **(B1)** and IL-1β **(B2)**. **(C)** CCL5 correlations with TNFα **(C1)** and IL-1β **(C2)**. RSEM, RNAseq by expectation-maximization.

Moreover, as TNFα and IL-1β are key inducers of CXCL8, CCL2 and CCL5 expression [e.g., (81)] we also determined the correlation between the expression levels of TNFα and IL-1β and each of the three chemokines in basal patients. The findings of Figure 2 indicate that the expression levels of TNFα and IL-1β were significantly correlated and coordinated with the presence of CXCL8, CCL2, and CCL5 in basal tumors.

### Pro-metastatic Chemokines Reach Their Highest Expression Levels When TNBC:Stroma “Contact” Co-cultures Are Stimulated by TNFα or IL-1β

**Figure 3:**
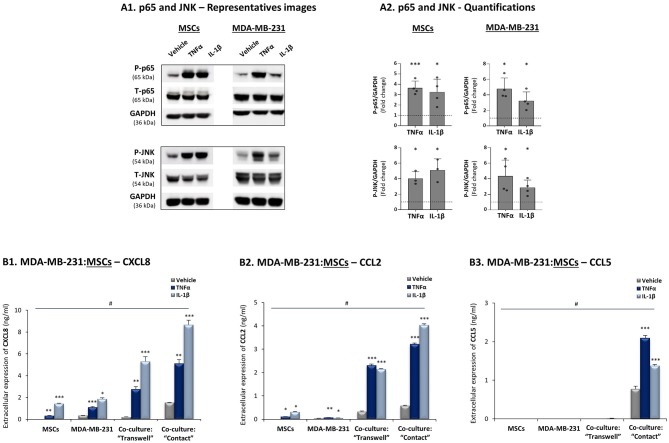
CXCL8, CCL2 and CCL5 reach their highest expression levels when TNBC:MSC “Contact” co-cultures are stimulated by TNFα or IL-1β. **(A)** Activation of p65 and JNK, determined by WB in MSCs and in human TNBC MDA-MB-231 cells upon 15 min stimulation by TNFα, IL-1β or vehicle control (based on kinetics and titration studies. Information on cytokine concentrations is given in “Materials and methods”). GAPDH was used as a loading control. **(A1)** A representative experiment and **(A2)** averages ± SD of p65 and JNK activation (fold induction) in *n* ≥ 3 independent experiments, performed with MSCs of 2 different donors. ***p < 0.001, *p ≤ 0.05 for differences between the values obtained for cytokine-stimulated cells and control non-stimulated cells. Dashed line stands for the value of 1 given to control cells. **(B)** CXCL8 **(B1)**, CCL2 **(B2)** and CCL5 **(B3)** expression levels were determined in TNBC:MSC co-cultures or each of the cell types grown alone, with or without TNFα or IL-1β stimulation. Co-cultures of MDA-MB-231 cells with MSCs were generated in “Transwell” and “Contact” conditions and were stimulated by TNFα (10 ng/ml), IL-1β (350 pg/ml) or vehicle control (similar for both cytokines) for 7 h. After change of media and growth for additional 60 h, the extracellular expression of CXCL8 in TNFα/IL-1β-free CM was determined by ELISA. ***p ≤ 0.001, **p ≤ 0.01, *p ≤ 0.05 for differences between TNFα- or IL-1β-stimulated cells and vehicle-treated cells, within each group. #*p*-values were <0.01 or <0.001 (in most cases) in comparisons of “Contact” co-cultures with all other treatments, as well as in comparisons of “Transwell” co-cultures with all other treatments. The results are of a representative experiment of *n* ≥ 3 independent experiments, performed with MSCs of 3 different donors.

To follow up on the above findings, we asked how the cytokines TNFα and IL-1β regulate TNBC:MSC interactions that may lead to elevated release of the chemokines CXCL8, CCL2 and CCL5. First, we validated that the two cytokines could activate transcription pathways that typically induce the expression of these chemokines, namely NF-κB/p65 and JNK/AP-1 (30, 67, 81, 82). Indeed, these pathways were rapidly activated by a brief TNFα and IL-1β stimulation of 15 min (time point and cytokine concentrations were determined by preliminary analyses) in the TNBC MDA-MB-231 cells and in MSCs (Figure 3A).

Then, to determine if CXCL8 is regulated by tumor-stroma-inflammation networks, MDA-MB-231:MSC co-cultures were plated in “Contact” conditions that enabled direct physical contacts between the two cell types or in “Transwell” conditions that allowed only for the exchange of soluble factors between tumor cells and stromal cells. These co-cultures and each cell type alone were stimulated by TNFα and IL-1β (or their vehicle) for 7 h (Cytokine concentrations were selected based on titration assays, as described in “Materials and methods”); the cytokines were removed and cytokine-free CM were collected 60 h later. These experiments revealed that the highest levels of CXCL8 were produced when “Contact” co-cultures were stimulated by TNFα and IL-1β (Figure 3B1), and that they were higher than in all other conditions including non-stimulated co-cultures and individual cell types treated with either cytokine. CXCL8 protein levels were elevated also in cytokine-stimulated “Transwell” conditions; however, their total levels (ng/ml) were significantly lower than in cytokine-stimulated “Contact” conditions (Figure 3B1). Accompanying qRT-PCR analyses indicated that CXCL8 induction was regulated at the transcription level (Supplementary Figure 1A), and that increased CXCL8 levels in cytokine-stimulated co-cultures did not result of elevated proliferation of the cells under these conditions (Supplementary Figure 2). Accordingly, we did not detect any substantial cell death or proliferation of any of the cell types due to cellular interactions or cytokine stimulation (Data not shown).

Additional experiments revealed similar regulatory modes for CCL2, demonstrating its highest expression levels in TNFα- and IL-1β-stimulated MDA-MB-231:MSC co-cultures, obtained partly in a contact-dependent process (Figure 3B2). With CCL5, absolute dependence on TNBC:MSC contacts was revealed, and its expression was further elevated when “Contact” co-cultures were stimulated by TNFα or IL-1β (Figure 3B3). As with CXCL8, TNFα and IL-1β induced the expression of CCL2 and CCL5 by elevating their mRNA levels (Supplementary Figures 1B,C).

**Figure 4:**
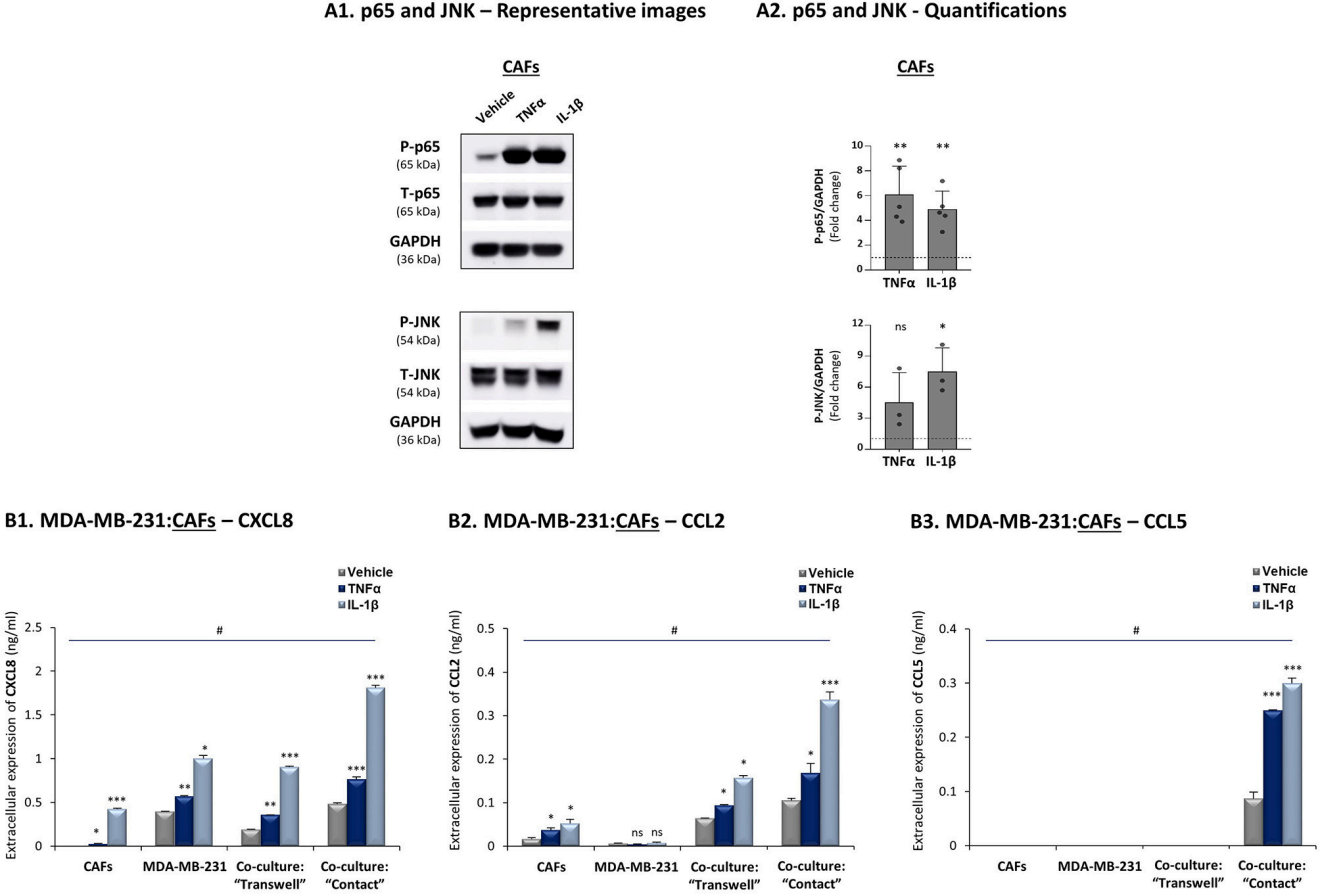
CXCL8, CCL2 and CCL5 reach their highest expression levels when TNBC:CAF “Contact” co-cultures are stimulated by TNFα and IL-1β. The Figure demonstrates similar experiments and statistical analyses as those of Figure 3, performed herein with MDA-MB-231 cells and CAFs, using similar cytokine concentrations. **(A)** Activation of p65 and JNK. **(B)** CXCL8 **(B1)**, CCL2 **(B2)** and CCL5 **(B3)** expression levels. In all parts of the Figure, the results are of a representative experiment of *n* ≥ 3 independent experiments. *, **, ***, # - As described in Figure 3. ns=non-significant.

Additional analyses performed with TNBC cells that interacted with breast cancer patient-derived CAFs revealed similar regulatory patterns to those described above with MDA-MB-231:MSC co-cultures: TNFα and IL-1β induced p65 and JNK activation in CAFs (Figure 4A) and elevated all three pro-metastatic chemokines to their highest levels of expression when tumor-stroma-inflammation interactions took place (Figure 4B); moreover, CXCL8 and CCL2 up-regulation depended partly on cell-to-cell contacts whereas induction of CCL5 was fully dependent on direct physical contacts between the tumor cells and the CAFs, and was further induced by stimulation with TNFα and IL-1β.

**Figure 5:**
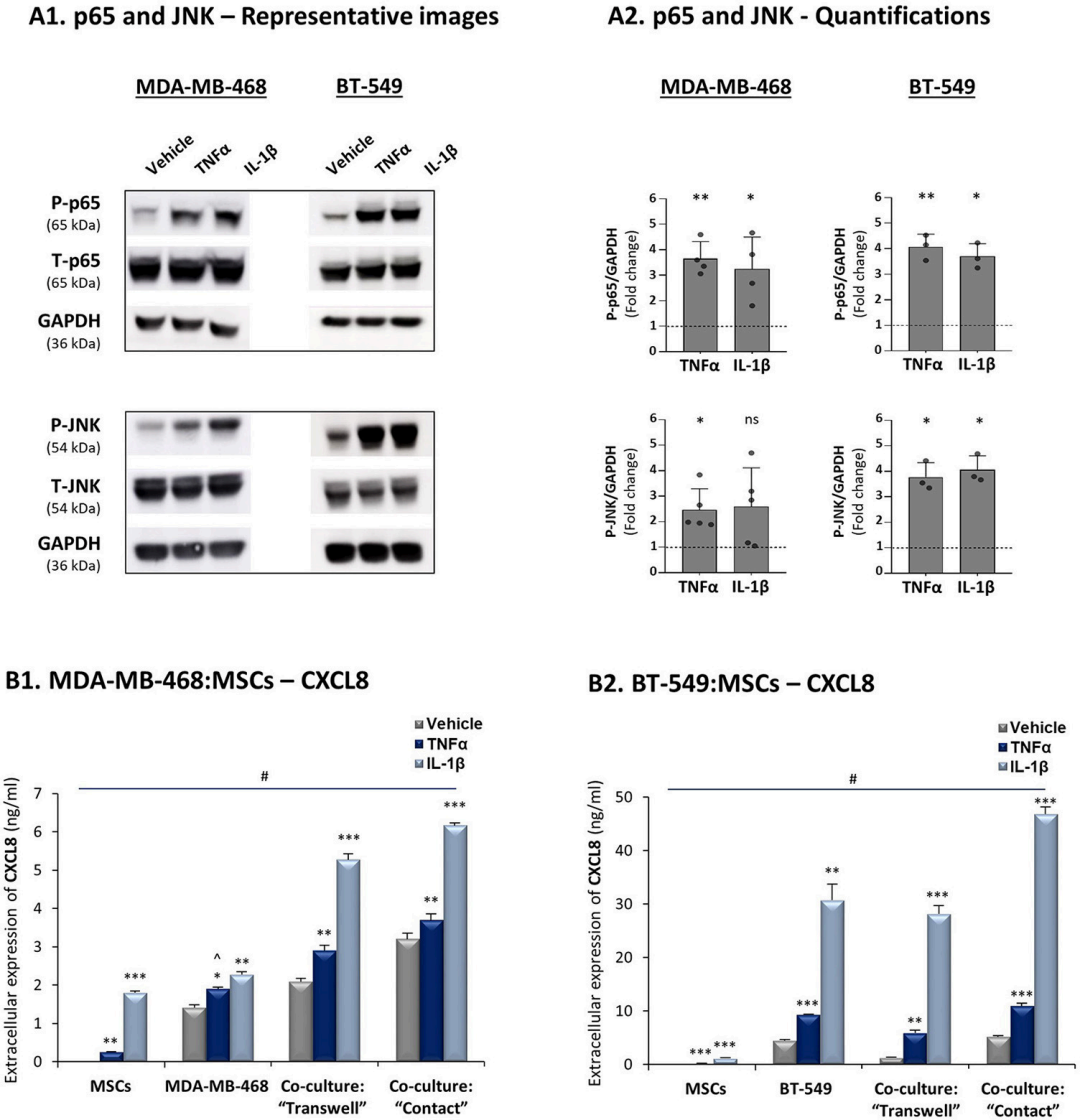
Increased production of CXCL8 is a general characteristic of tumor-stroma-inflammation networks established with TNBC cells. The Figure demonstrates similar experiments and statistical analyses as those of Figure 3, performed herein with human TNBC MDA-MB-468, BT-549 cells and MSCs. Cytokine concentrations: MDA-MB-468 cells - 50 ng/ml TNFα and 500 pg/ml IL-1β; BT-549 cells - 25 ng/ml TNFα and 350 pg/ml IL-1β. **(A)** Activation of p65 and JNK. **(B)** CXCL8 expression levels. In all parts of the Figure, the results are of a representative experiment of *n* ≥ 3 independent experiments, performed with MSCs of 2 different donors. ^∧^In panel 5B1, this value was significant in 2 out of 3 experiments. *, **, ***, # - As described in Figure 3. ns=non-significant.

In view of the high heterogeneity of TNBC tumors (4), we asked if similar regulatory patterns exist in co-cultures of other human TNBC cells - MDA-MB-468 and BT-549 - with MSCs. The findings of Figure 5A indicate that in both cell lines TNFα and IL-1β induced p65 and JNK activation and that the highest CXCL8 expression levels were produced when these TNBC cells physically interacted with MSCs in the presence of TNFα and IL-1β (Figure 5B). Of interest, the elevation in CXCL8 levels following cytokine stimulation of MDA-MB-468:MSC co-cultures partly depended on physical contacts between the two cell types (Figure 5B1), as was seen in MDA-MB-231:MSC co-cultures (Figure 3B1). In parallel, in BT-549:MSC co-cultures, CXCL8 elevation in IL-1β-stimulated cells depended on cell-to-cell contacts, while TNFα did not have much of an impact under “Contact” conditions (Figure 5B2).

**Figure 6:**
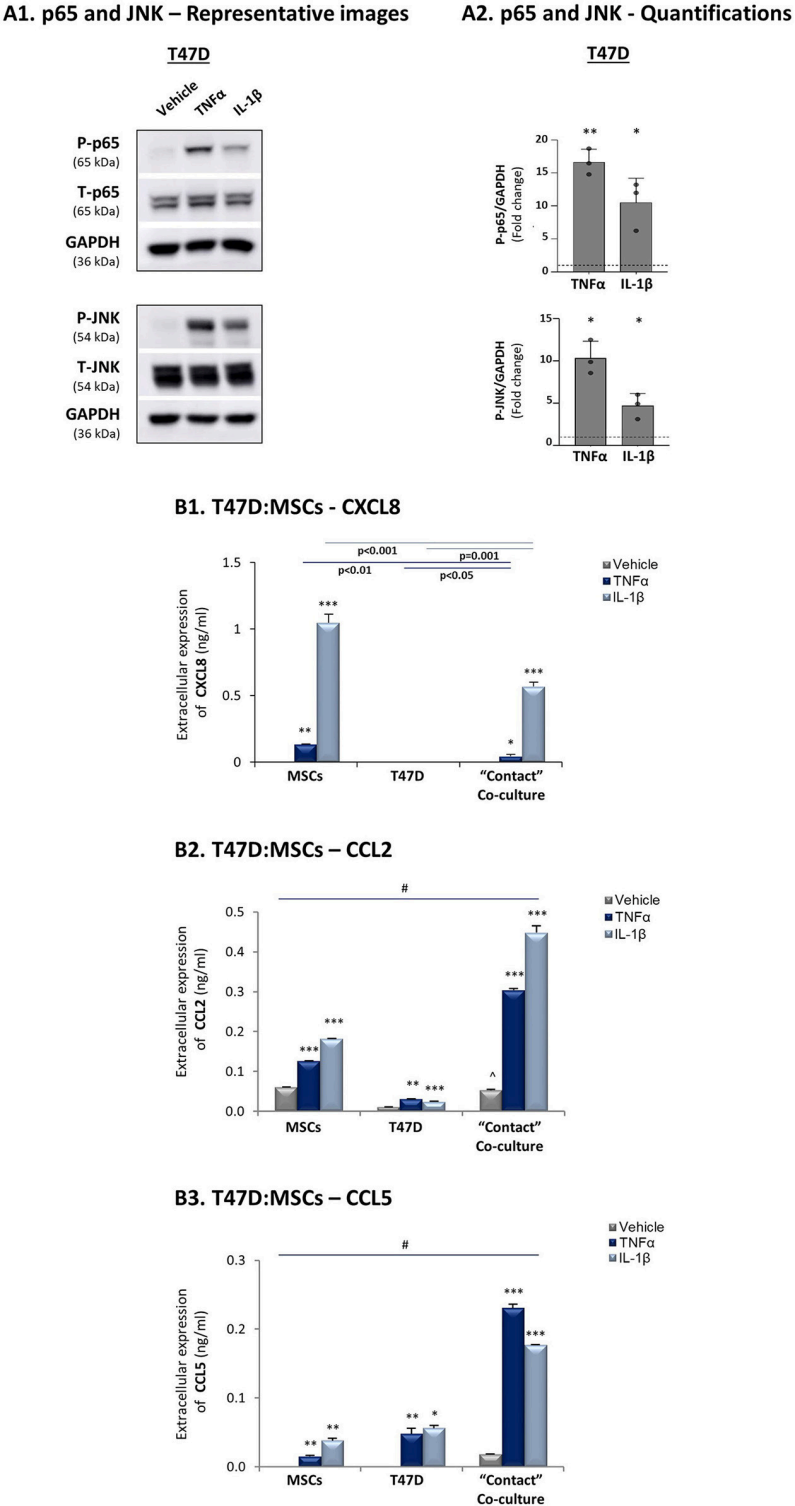
CXCL8 expression is down-regulated in TNFα- and IL-1β-stimulated luminal-A (T47D): MSC “Contact” co-cultures. The Figure demonstrates similar experiments and statistical analyses as those of Figure 3, performed herein with T47D cells and MSCs. Cytokine concentrations: 50 ng/ml TNFα and 500 pg/ml IL-1β. **(A)** Activation of p65 and JNK. **(B)** CXCL8 **(B1)**, CCL2 **(B2)** and CCL5 **(B3)** expression levels. ^∧^In panel B2, comparisons to non-stimulated MSCs were non-reproducible. In all parts of the Figure, the results are of a representative experiment of *n* = 3 independent experiments, performed with MSCs of ≥2 different donors. *, **, ***, # - As described in Figure 3.

**Figure 7:**
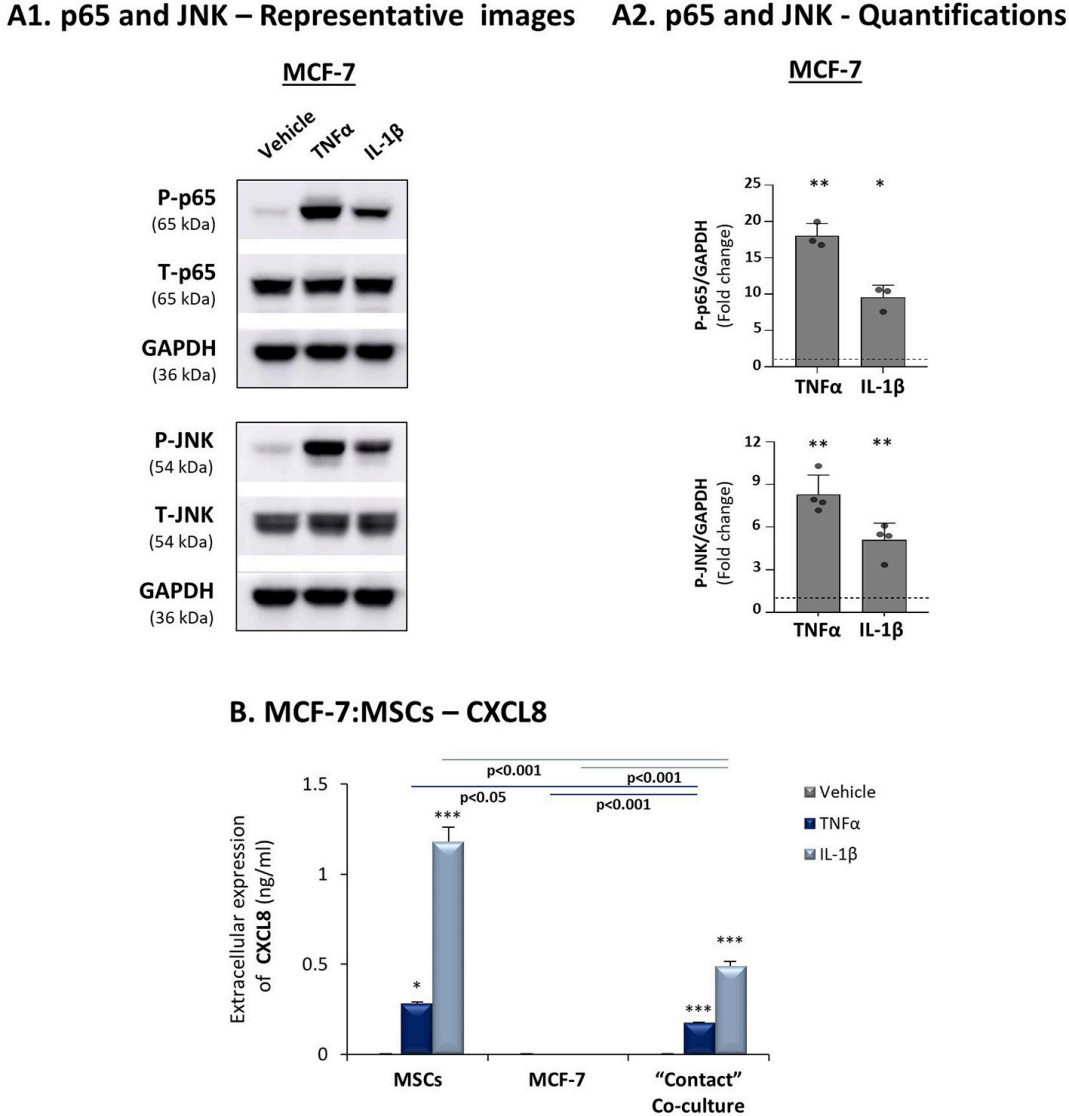
CXCL8 expression is down-regulated in TNFα- and IL-1β-stimulated luminal-A (MCF-7): MSC “Contact” co-cultures. The Figure demonstrates similar experiments and statistical analyses as those of Figure 3, performed herein with MCF-7 cells and MSCs. Cytokine concentrations: 50 ng/ml TNFα and 500 pg/ml IL-1β. **(A)** Activation of p65 and JNK. **(B)** CXCL8 expression levels. In all parts of the Figure, the results are of a representative experiment of n≥3 independent experiments, performed with MSCs of 2 different donors. *, **, ***, # - As described in Figure 3.

To follow up on the data of Figure 1, indicating that the expression levels of the pro-inflammatory cytokines TNFα and IL-1β and of their targets - CXCL8, CCL2 and CCL5 - were significantly lower in luminal-A patients than in basal patients, we determined how these three chemokines are affected by TNFα and IL-1β stimulation of luminal-A:MSC co-cultures. Despite the fact that TNFα and IL-1β induced potent p65 and JNK activation in T47D and MCF-7 luminal-A cells (Figures 6A, 7A), CXCL8 levels were not increased but rather were decreased when the tumor cells interacted with MSCs in the presence of TNFα and IL-1β (Figures 6B1, 7B). In parallel, CCL2 and CCL5 levels were increased by TNFα- and IL-1β-stimulated T47D:MSC “Contact” co-cultures (Figures 6B2,B3), but their expression levels were, in general, much lower than those obtained by TNFα- and IL-1β-stimulated TNBC:MSC co-cultures (Figures 3B2,B3). Further studies with MCF-7 luminal-A cells demonstrated elevations in the expression of CCL2 and CCL5 in some of the assays under “Contact” co-culture conditions following TNFα and IL-1β stimulation; however, the expression levels of these chemokines were often too low to provide clear-cut results (Data not shown).

These studies were followed by analyses of additional pro-metastatic effects, including tumor cell migration, invasion and angiogenesis. To this end, in the TNBC part we focused on the MDA-MB-231 cells because of their high metastatic potential. In luminal-A studies we chose to investigate MCF-7 cells because of our previous research indicating that they expressed more robust metastasis-related properties than T47D cells when stimulated by pro-inflammatory signals; additional investigations by our group also indicated that MCF-7 cells responded vigorously to TNFα-containing TME signals in *in vivo* metastasis studies (83–86).

### Tumor-Stroma-Inflammation Networks Established With TNBC Cells Lead to Elevated Angiogenesis

**Figure 8:**
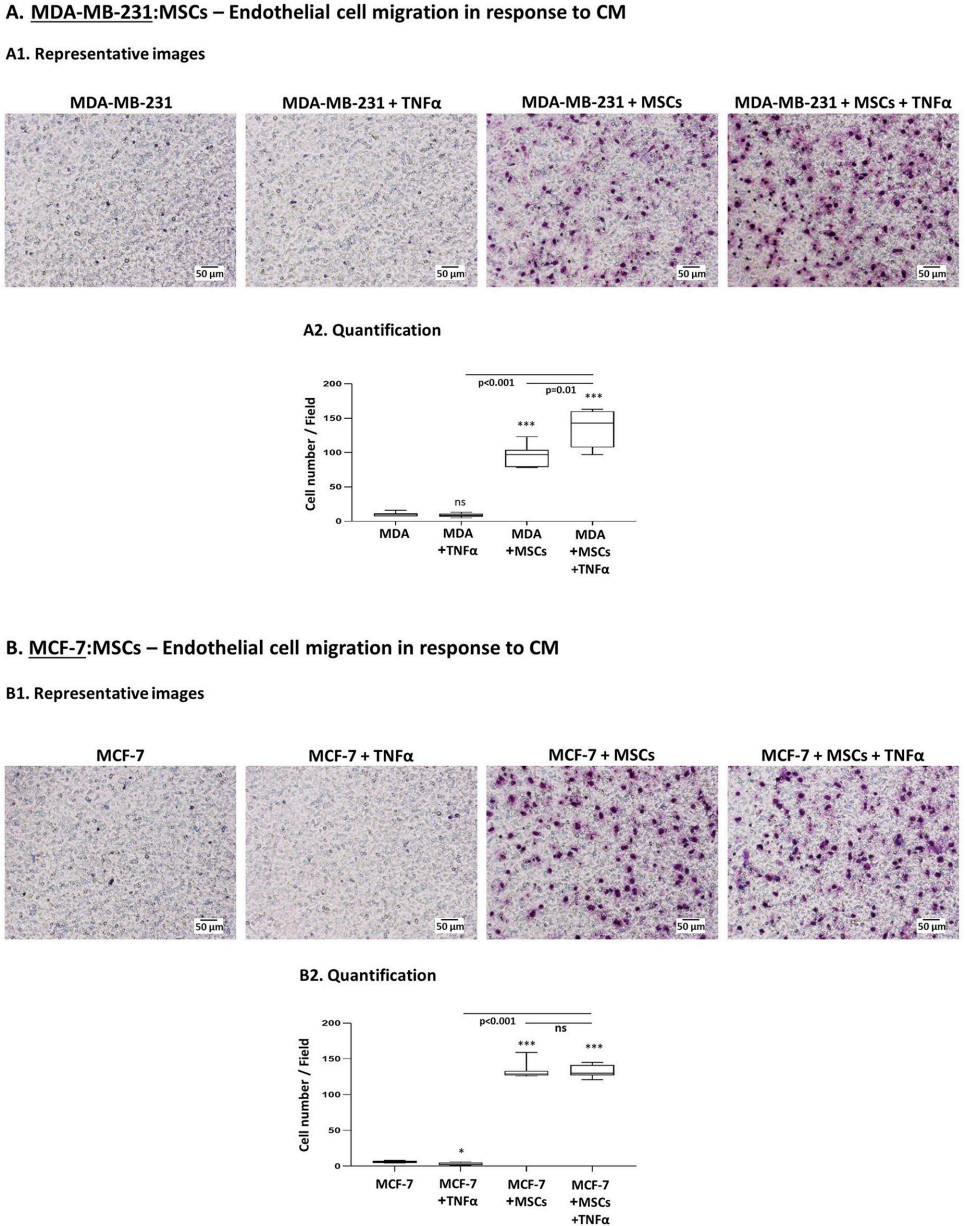
The pro-angiogenic activities of factors released by tumor:MSC “Contact” co-cultures are promoted by TNFα in TNBC but not in luminal-A cells. Studies of endothelial cell (HPMEC) migration in response to CM derived from different cell combinations of MDA-MB-231 cells (“MDA”) **(A)** and MCF-7 cells **(B)**. **(A1, B1)** Representative photos of HPMEC migration in response to TNFα-free CM derived from TNFα-stimulated tumor:MSC “Contact” co-cultures (10 ng/ml), from tumor cells alone, from tumor cells stimulated by TNFα alone and from tumor cells grown under “Contact” conditions with MSCs only. Bars, 50 μm. **(A2, B2)** Migrated HPMEC were counted in multiple photos per insert of the experiments presented in A1 and B1. ***p < 0.001, *p < 0.05, ns=non-significant for comparisons between CM of different cell combinations and CM of tumor cells treated by vehicle. Photos and their quantifications are representatives of *n* ≥ 3 independent experiments, performed with MSCs of 3 different donors.

To determine the functional consequences of tumor-stroma-inflammation networks, we first determined the ability of factors released by TNBC:MSC co-cultures stimulated by TNFα to promote processes involved in angiogenesis. Here, endothelial cells (HPMEC) sprouting assays demonstrated conclusive evidence to higher angiogenesis-supporting potential of TNFα-free CM derived from TNFα-stimulated “Contact” MDA-MB-231:MSC (Supplementary Figure 3) compared to CM obtained from control MDA-MB-231 cells. However, these studies did not reveal concrete information on the angiogenic potential of CM obtained from tumor cells + MSCs or from tumor cells + TNFα. Thus, additional studies were designed to provide another level of information on the ability of CM derived from the different groups to induce the migration of HPMEC in response to CM derived from different conditions. This assay enabled us to clearly demonstrate that the highest levels of endothelial cell migration were achieved when MDA-MB-231 cells interacted with MSCs in the presence of TNFα (Figure 8A). Moreover, our findings emphasized the contribution of TNFα to the angiogenic potential revealed by tumor cells grown in the presence of stromal cells.

We then performed parallel studies with luminal-A MCF-7 cells and found that CM of “Contact” MCF-7:MSC co-cultures had strong angiogenic activities as with TNBC cells (Figure 8B); however, in contrast to our studies with MDA-MB-231 cells (Figure 8A), in studies of MCF-7 cells TNFα did not push the angiogenic response induced by CM of “Contact” co-cultures any further.

### Tumor-Stroma-Inflammation Networks Promote the Migratory and Invasive Properties of TNBC Cells

**Figure 9:**
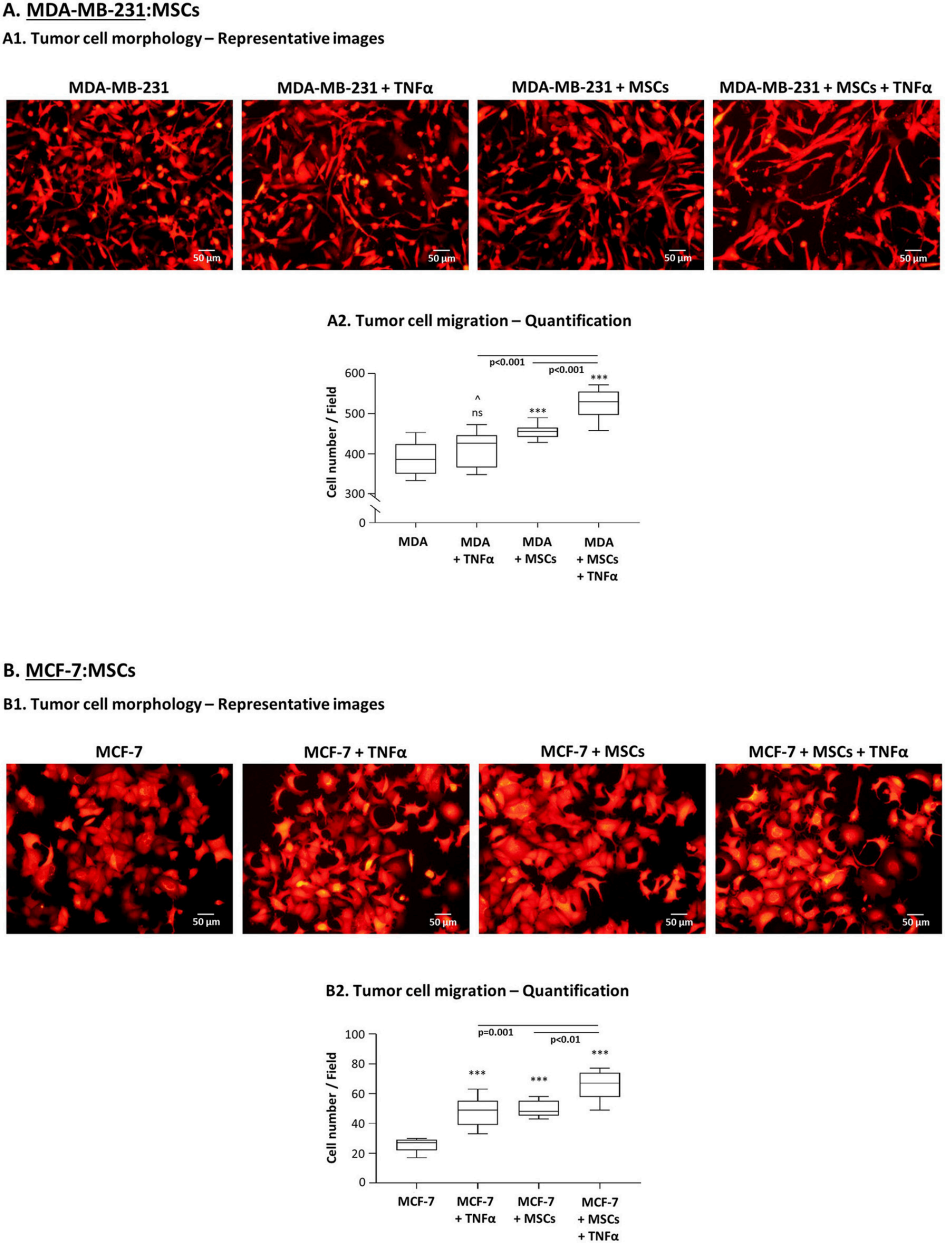
TNBC cells, and less so luminal-A cells, acquire migration-related characteristics upon “Contact” co-culturing with MSCs in the presence of TNFα. **(A)** Morphology and migration phenotypes of MDA-MB-231 cells (“MDA”). **(A1)** Morphology of mCherry-MDA-MB-231 cells grown with MSCs in the presence of TNFα (10 ng/ml), compared to tumor cells treated by vehicle, tumor cells stimulated by TNFα or tumor cells grown with MSCs only. Bar, 50 μm. **(A2)** Migration of mCherry-MDA-MB-231 cells (“MDA”) grown in “Contact” co-cultures with MSCs in the presence of TNFα (10 ng/ml) compared to migration of MDA-MB-231 cells treated by vehicle only, of MDA-MB-231 cells stimulated by TNFα or of MDA-MB-231 cells grown in co-culture with MSCs only. Migration assays were performed in response to medium containing 10% FBS, for 12 h. ***p < 0.001, ns=non-significant for differences between migration of tumor cells in different combinations, compared to the migration of non-stimulated TNBC cells. ^∧^In panel **(A2)**, this value was significant in 1 out of 3 experiments. Representative fluorescent photos of migrating cells are presented in Supplementary Figure 4. In all sections of Part **(A)**, the Figures demonstrate representative experiments of *n* = 3 independent experiments of each type, performed with MSCs of ≥2 different donors. **(B)** Morphology and migration phenotypes of MCF-7 cells, determined as described in Part **(A)**, unless otherwise indicated. **(B1)** Morphology of mCherry-MCF-7 cells. Bar, 50 μm. **(B2)** Migration of Hoechst-loaded MCF-7 cells was performed in response to medium containing 10% FBS for 21 h through fibronectin-coated membranes, in similar combinations as of MDA-MB-231 cells in Part **(A)** (TNFα: 10 ng/ml). ***p < 0.001; Representative photos of migrating cells are presented in Supplementary Figure 5. In all sections of Part **(B)**, the Figures demonstrate representative experiments of *n* ≥ 3 independent experiments, performed with MSCs of ≥2 different donors.

Next, we determined the effects of the tumor-stroma-inflammation network on tumor cell morphology, migration and invasion. First, we found that in TNFα-stimulated MDA-MB-231:MSC “Contact” co-cultures, the tumor cells acquired very elongated morphology (Figure 9A1) which is typical of cells that express high motility capabilities (87, 88). The morphology of the tumor cells under this condition was robustly different from the morphology of tumor cells grown alone, of tumor cells stimulated by TNFα and of tumor cells grown with MSCs only (Figure 9A1). These studies were followed by migration assays of MDA-MB-231 cells, known as having an aggressive phenotype which is manifested by a relatively high basal migratory potential. Despite their high basal motility, the interactions of MDA-MB-231 cells with MSCs in the presence of TNFα have led to significantly higher migratory capacity of the tumor cells compared to tumor cells grown alone (Figure 9A2). Of note, MDA-MB-231 cells grown in the presence of MSCs only or with TNFα alone did not migrate as well as tumor cells grown with MSCs and TNFα (Figure 9A2). Supplementary Figure 4 demonstrates representative photos of MDA-MB-231 cells that migrated in the different study groups, identified by fluorescent staining.

In parallel, experiments performed with MCF-7 cells indicated that their morphology was modified by TNFα stimulation (Figure 9B1) toward a metastasis-relevant phenotype [in line with our findings in (83–85)]. MCF-7 cells that grew in contact with MSCs also demonstrated modifications in their morphology, different than those induced by TNFα stimulation. However, in contrast to our findings with MDA-MB-231 cells (Figure 9A1), when TNFα was added to MCF-7:MSC “Contact” co-cultures, no additivity was found between TNFα and the MSCs in inducing more robust morphological changes in the tumor cells (Figure 9B1). Of note, the TNFα-stimulated MCF-7 cells that grew in co-culture with MSCs acquired elevated migratory capacity compared to control cells (Figure 9B2; Supplementary Figure 5). However, the overall migratory potential of MCF-7 cells at the tumor-stroma-inflammation setting (Figure 9B2) was much lower of MDA-MB-231 cells (Figure 9A2), although the two cell types were plated in same numbers in migration transwells. The relatively low migratory capacities of MCF-7 cells were noted despite the fact that they were given the proper conditions to support their migration (fibronectin coating of membranes and longer migration time than MDA-MB-231 cells).

**Figure 10:**
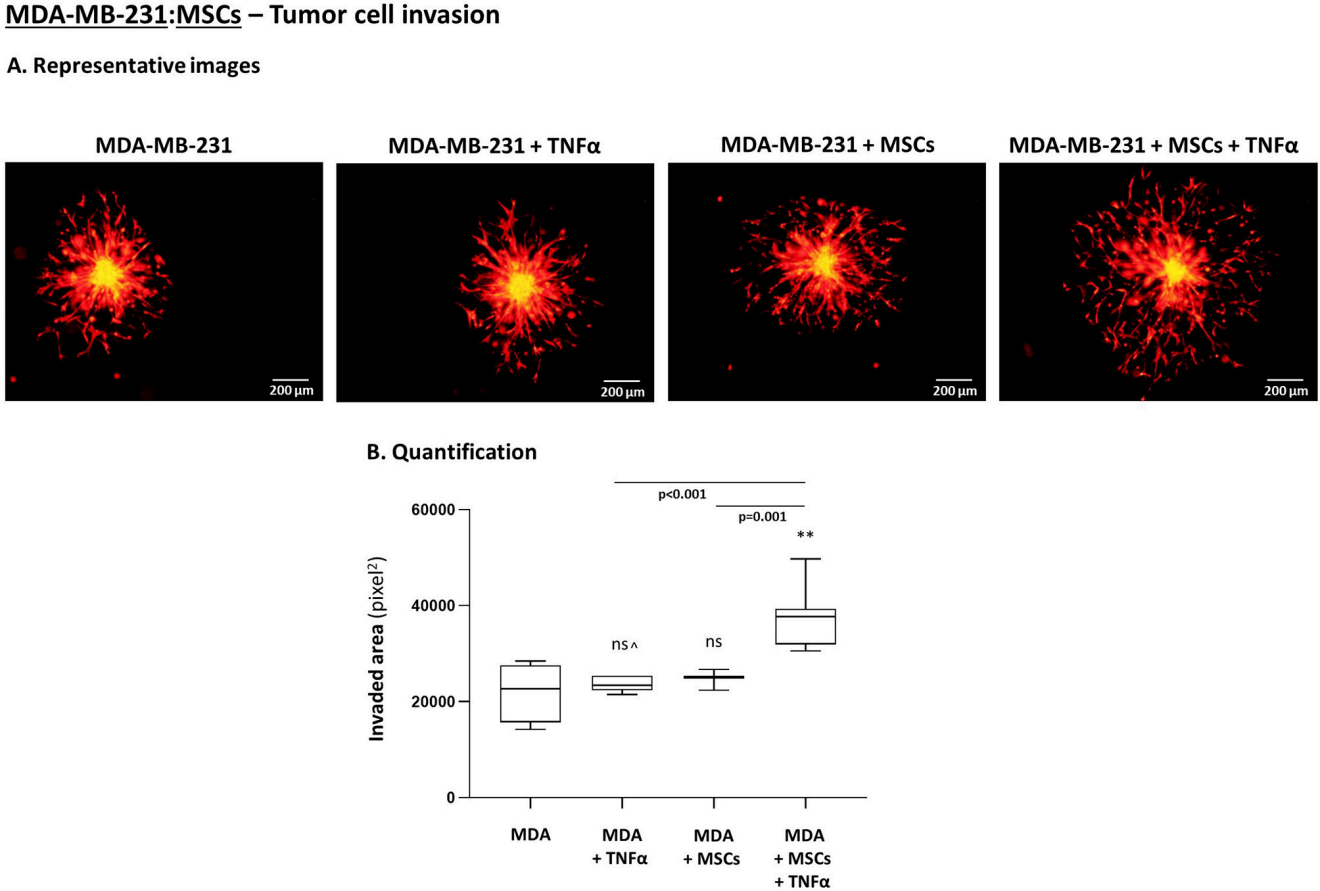
TNBC cells acquire elevated invasive properties upon “Contact” co-culturing with MSCs in the presence of TNFα. The Figure demonstrates tumor cell invasion out of 3D spheroids that were formed by mCherry-MDA-MB-231 cells (“MDA”) alone or by tumor cells in “Contact” co-culturing with MSCs. The spheroids were imbedded into matrigel and then stimulated by TNFα (10 ng/ml) or vehicle for 48 h. **(A)** Representative photos. Bar, 200 μm. **(B)** Invasion was quantified in multiple spheroids by ImageJ. **p < 0.01, ns=non-significant for differences between TNBC cell invaded out of spheroids in different combinations, compared to the invasion of non-stimulated TNBC-only spheroids. ^∧^See “Note” in legend to Figure 11. Photos and their quantifications are representatives of *n* > 3 independent experiments, performed with MSCs of 2 different donors.

**Figure 11:**
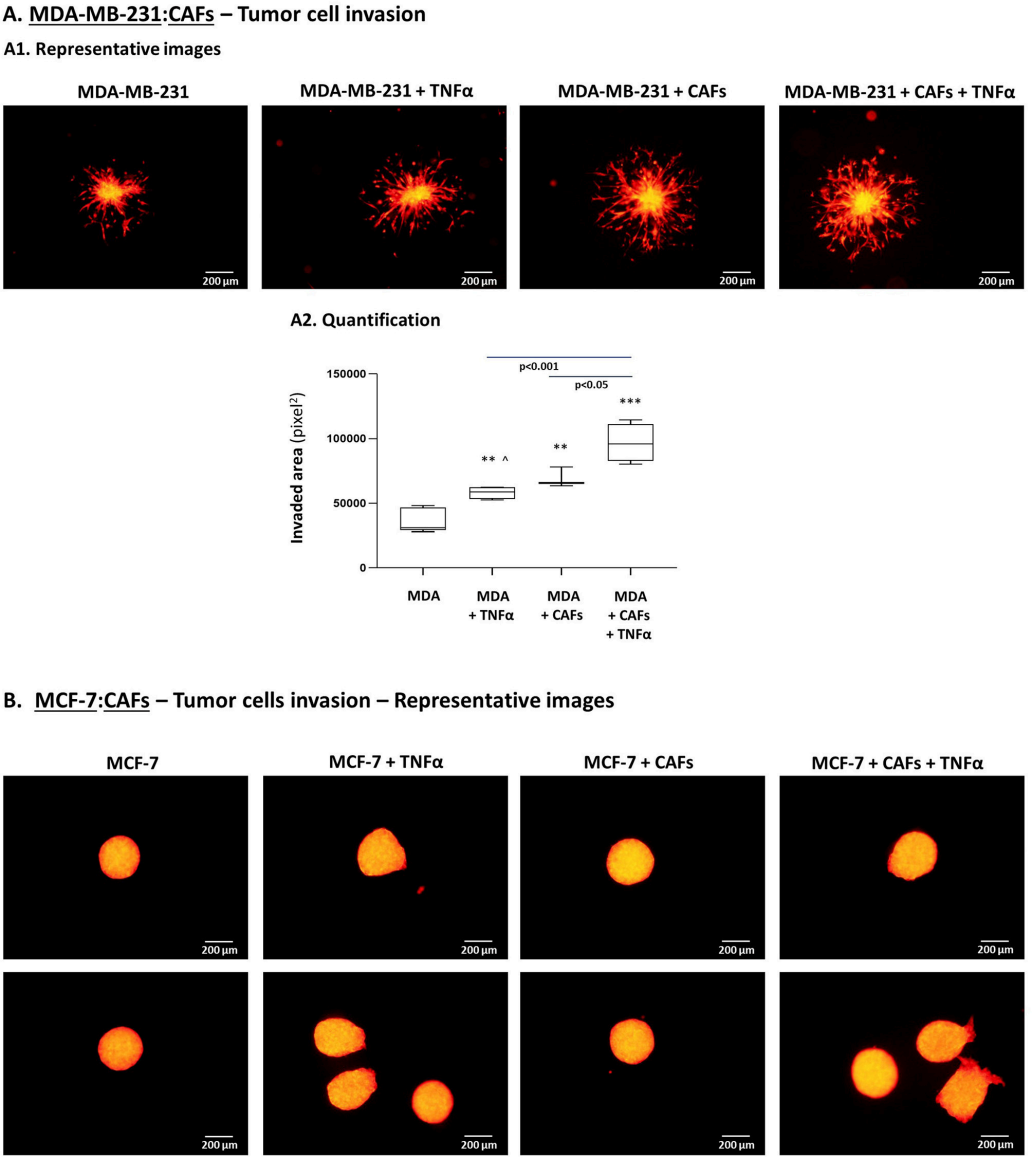
TNBC cells but not luminal-A cells acquire elevated invasive properties upon “Contact” co-culturing with patient-derived CAFs in the presence of TNFα. **(A)** The Figure demonstrates similar experimental setup as in Figure 10, performed herein with mCherry-MDA-MB-231 cells (“MDA”) and CAFs, using similar cytokine concentrations. [^∧^Note: Possibly due to technical reasons (different matrigel batches) TNFα stimulation elevated tumor cell invasion in this setting but not in Figure 10]. **(A1)** Representative photos. Bar, 200 μm. **(A2)** Invasion was quantified in multiple spheroids by ImageJ. ***p < 0.001, **p < 0.01 for differences between invasion of tumor cells in different combinations, compared to the invasion of TNBC cells grown alone in spheroids. Photos and their quantifications are representatives of *n* > 3 independent experiments. **(B)** MCF-7 cells have undergone similar procedures to those described in Figure 10, for 96 h (TNFα 10 ng/ml). Because invasion of MCF-7 cells out of the spheroids was minimal or absent, quantitation could not be performed. Instead, two representative photos out of many taken in *n* > 3 independent experiments, are provided for each treatment. Bar, 200 μm.

To follow up on these findings, we investigated the ability of TNBC cells to invade out of 3D spheroids, a process requiring migration and invasion through extracellular proteins. We noted significantly increased invasion of MDA-MB-231 cells when they interacted with MSCs in the presence of TNFα stimulation (Figure 10), compared to all other cell combinations. Moreover, MDA-MB-231 cells exerted significantly increased invasion also when they interacted with patient-derived CAFs in the context of TNFα (Figure 11A; Please see “Note” in the legend of Figure 11A). In contrast to the TNBC cells, MCF-7 luminal-A cells that interacted with patient-derived CAFs in the presence of TNFα demonstrated very minor, if any, invasive properties (Figure 11B), even after longer invasion time compared to MDA-MB-231 cells (96 h for MCF-7 cells; 48 h for MDA-MB-231 cells).

### TNBC-Stroma-Inflammation Networks Lead Through CXCL8 Activities to Increased Angiogenesis, as Well as to Elevated Migration and Invasion of TNBC Cells

The findings demonstrated so far indicated that tumor-stroma-inflammation networks can lead in TNBC to (1) increased production of pro-metastatic chemokines such as CXCL8 and (2) elevated angiogenesis, tumor cell migration and invasion. To connect between these two processes, we asked if CXCL8 - selected because of its robust pro-angiogenic activities and pro-metastatic effects at the levels of TME and the tumor cells alike - was involved in mediating the functional properties of TNBC cells when the tumor-stroma-inflammation network was established.

**Figure 12:**
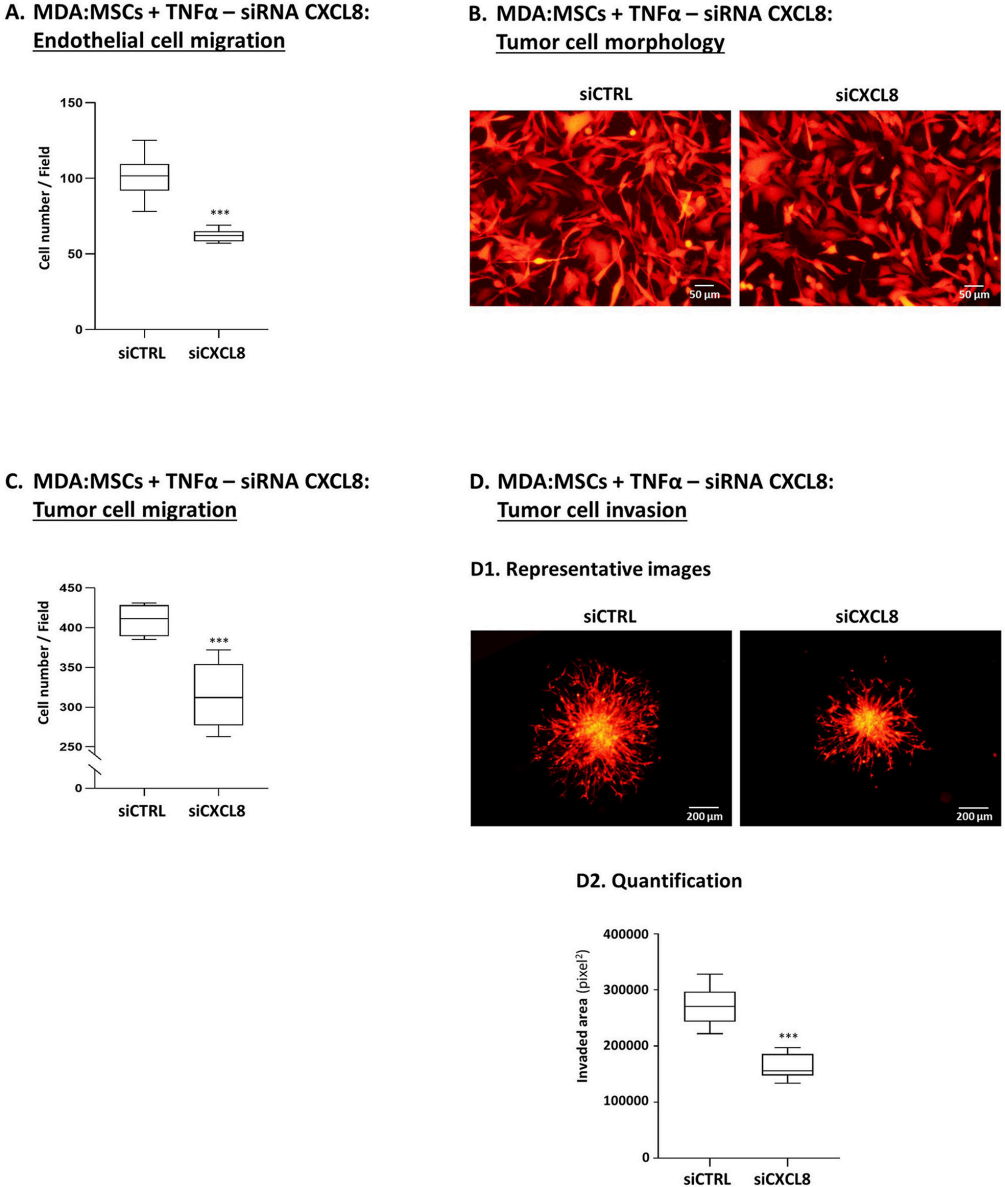
Tumor-stroma-inflammation networks lead through CXCL8 activities to increased angiogenesis, invasion-related tumor cell morphology, tumor cell migration and invasion in TNBC cells. MDA-MB-231 cells (“MDA”) and MSCs were both transfected by siCXCL8 or siCTRL. Parallel studies indicated that the efficiency of CXCL8 down-regulation was high [80–90% in most experiments, as in (80); Data not shown]. The cells were grown in “Contact” co-cultures and were stimulated by TNFα (10 ng/ml); then, the cells or their CM were assayed in the following tests: **(A)** Endothelial cell migration (Procedures as in Figure 8A). ***p < 0.001; **(B)** Tumor cell morphology (Procedures as in Figure 9A1). Bar, 50 μm; **(C)** Tumor cell migration (Procedures as in Figure 9A2). ***p < 0.001; **(D)** Tumor cell invasion (Procedures as in Figure 10). **(D1)** Representative photos. Bar, 200 μm. **(D2)** Quantification. ***p < 0.001. In all parts of the Figure, photos and their quantifications are representatives of *n* = 3 independent experiments, performed with MSCs of 2 different donors.

To this end, we generated TNFα-stimulated MDA-MB-231:MSC “Contact” co-cultures in which CXCL8 was down-regulated in high efficiency by siRNA in the tumor cells and in the MSCs simultaneously [80–90% efficiency was found in CXCL8 down-regulation by the siRNA, similar to our findings in our parallel study (80); that study also demonstrates which of the cells contributed more to CXCL8 production when the tumor-stroma-inflammation network was established with MDA-MB-231 cells]. The findings of Figure 12 clearly indicate that CXCL8 played significant roles in driving forward all metastasis-related alterations that were induced by the tumor-stroma-inflammation network in TNBC. Here, we found that in the absence of CXCL8 expression, endothelial cell migration in response to CM of TNFα-stimulated MDA-MB-231:MSC co-cultures was significantly reduced (Figure 12A). Moreover, upon CXCL8 down-regulation, the migration-relevant morphology of the tumor cells was partly reversed (Figure 12B), and the migration and particularly the invasion potentials of the tumor cells were significantly reduced (Figures 12C,D).

### Tumor-Stroma-Inflammation Networks Promote the *in vivo* Pro-metastatic Properties of TNBC Cells

Many published studies have described the ability of MSCs and CAFs to promote the metastatic phenotype of TNBC cells (12, 16–18, 21, 23, 26, 65). Yet, they have not directly determined the impact of the pro-inflammatory signals on tumor growth and metastasis when TNBC:stroma interactions are established. Our above findings motivated us to determine whether the pro-inflammatory signals delivered by TNFα to TNBC:MSC “Contact” co-cultures *in vitro* would potentiate tumor growth or metastasis in an animal model system.

**Figure 13:**
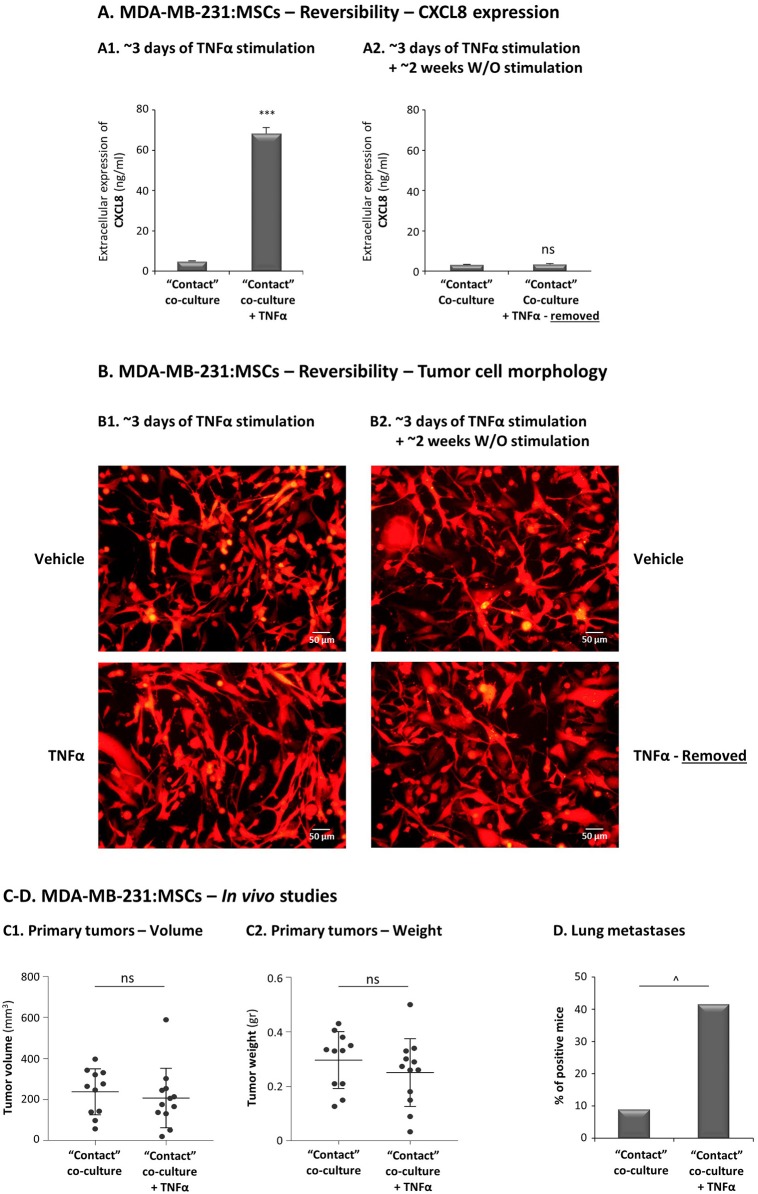
TNFα promotes the metastatic potential of TNBC cells grown in contact with MSCs. **(A,B)** Reversibility of TNFα-induced tumor-promoting phenotypes, generated *in vitro* in “Contact” MDA-MB-231:MSC co-cultures following TNFα removal. mCherry-MDA-MB-231 cells were co-cultured in “Contact” conditions with MSCs in the presence of TNFα (10 ng/ml) or vehicle control for 67 h (termed “~3 days of TNFα stimulation") **(A1,B1)**. Then, vehicle-treated and TNFα-stimulated MDA-MB-231:MSCs co-cultures were re-cultured without further TNFα stimulation for additional 10–14 days (termed “~3 days of stimulation + ~2 weeks W/O TNFα stimulation) **(A2,B2)**. At both time points (~3 days and ~2 weeks), extracellular CXCL8 levels were determined in cell supernatants by ELISA **(A)** and tumor cell morphology was determined by fluorescent microscopy **(B)**. ***p < 0.001, ns, not significant. Bar, 50 μm. In all panels of section **(A,B)**, the results are representatives of *n* = 3 independent experiments, performed with MSCs of 2 different donors. **(C,D)** mCherry-MDA-MB-231 cells were grown in “Contact” co-cultures with MSCs in the presence of TNFα (10 ng/ml) or vehicle control, for 67 h (~3 days). Then, co-cultured cells were injected to the mammary fat pad of nude mice in 2 independent experiments (For additional experimental details please see “Materials and methods”). Total mice numbers were: (1) In the group of mice administered with TNFα-stimulated co-cultures: *n* = 12; (2) In the group of mice administered with vehicle-exposed co-cultures: *n* = 11. At the end of the experiment (~30 days post injection), primary tumor size was determined by volume **(C1)** and weight **(C2)**. ns=non-significant. **(D)** Metastases in lungs were detected by mCherry signals using the Cri Maestro fluorescence imaging system. ^∧^*p* = 0.095.

The ability of TNFα to potentiate the *in vivo* aggressiveness of TNBC cells grown with MSCs requires that the *in vitro* advantages given to the tumor cells by their 3-day exposure to MSCs and to the cytokine, will persist *in vivo* (the cytokine is removed prior to injection to mice); Thus, we first determined whether the increased pro-metastatic capabilities endowed on the tumor cells by their co-culturing with stromal cells in the presence of TNFα withhold when TNFα is removed. To this end, TNBC:MSC “Contact” co-cultures were established for 67 h with TNFα stimulation, leading to high CXCL8 levels and clear changes in tumor cell morphology (Figures 13A,B); then, TNFα was removed and the growth of TNBC:MSC “Contact” co-cultures was continued in TNFα-deprived medium for ~2 weeks. The findings of Figure 13 demonstrate that the effects of TNFα were reversible: ~2 weeks after TNFα removal the elevation in CXCL8 was completely abolished (Figure 13A) and the elongated cell morphology was almost entirely diminished (Figure 13B).

Taking into account these findings on reversibility of TNFα-mediated effects, proposing that the benefits that were provided by TNFα stimulation to TNBC:MSC co-cultures may not persist *in vivo*, we proceeded to the animal system setting. Here, we introduced an experimental design that will enable us to directly assess the impacts of TNFα on the metastatic potential of TNBC cells when they interacted with stromal cells. To this end, MDA-MB-231 cells were grown in “Contact” co-cultures with MSCs in the presence of TNFα for 67 h, and were compared to vehicle-exposed MDA-MB-231:MSC cultures, serving as controls. The cells were then administered to the mammary fat pads of female mice. To strengthen *in vivo* the possible effects of TNFα when it acted on MDA-MB-231 and MSC “Contact” co-cultures, CM containing factors released by TNFα-stimulated MDA-MB-231:MSC “Contact” co-cultures (but deprived of TNFα itself) were injected in proximity to tumors generated by TNFα-stimulated co-cultures; in parallel, control media were administered to tumors arising from injection of vehicle-treated co-cultures. The findings of Figure 13C indicate that the sizes and weights of primary tumors were similar in the two groups of mice; however, most importantly, the metastatic potential of the tumor cells that interacted with MSCs was increased by in-culture TNFα stimulation (Figure 13D). This effect was revealed by the elevated incidence of mice carrying lung metastases following MDA-MB-231 co-culturing with MSC under the influence of TNFα *in vitro*, compared to the control group in which the co-cultured cells were not exposed to TNFα (Figure 13D).

## Discussion

The fundamental roles of the TME in promoting cancer progression are now well-appreciated, with stromal cells and pro-inflammatory elements being key contributors to disease development and metastasis. In the complex milieu that exists in tumors, cross-talks between the different TME players and the tumor cells eventually establish intricate networks whose roles in dictating disease course are still poorly defined and characterized.

In the present study, we were particularly interested in elucidating the roles of tumor-stroma-inflammation networks in regulating tumor progression in TNBC, a most aggressive subtype of breast cancer. In our study, we have used potent and most clinically relevant pro-inflammatory cytokines - TNFα and IL-1β - that are expressed in breast tumors and have pro-metastatic functions in TNBC. Our study provides novel findings indicating that interactions between TNBC cells and MSCs/CAFs in the presence of such pro-inflammatory cytokines can lead to significantly enhanced pro-metastatic phenotypes of the TME and of the tumor cells themselves. This was illustrated by increased expression of pro-metastatic chemokines, by elevated ability to induce angiogenesis, as well as by higher migratory and invasive capabilities of the tumor cells. Ultimately, the end result of the activities of the tumor-stroma-inflammation network was a higher metastatic potential of TNBC cells *in vivo*.

The tumor-stroma-inflammation network was found in our study to strongly induce the expression of the pro-metastatic chemokines CXCL8, CCL2 and CCL5. These chemokines are pro-inflammatory factors and as such contribute to cancer inflammation by recruiting myeloid inflammatory cells, as well as immune-suppressive cells, to tumors and metastases (27, 28, 31, 35, 89). In addition, of the three chemokines, particularly CXCL8 but also CCL2, are potent angiogenic factors that contribute to TNBC progression (90–93). Moreover, direct activities of the chemokines on tumor cells have led to increased invasion in TNBC cells (94–96).

As part of their pro-metastatic roles in TNBC, CXCL8, CCL2 and CCL5 and their receptors - for example, CXCR2 for CXCL8 and CCR2 for CCL2 - contributed to the pro-tumorigenic activities of stromal cells in TNBC mouse model systems (19–26). MSC/fibroblast-derived chemokines, including murine CXCL1 and CXCL2 (counterparts of human CXCL8), CCL2 and CCL5 were associated with recruitment of neutrophils, tumor-associated macrophages and myeloid-derived suppressor cells to TNBC tumors, where they promoted disease course (22, 25, 63, 65). CXCL8 and CCL5, produced by bone marrow- and adipose-derived MSCs were prime inducers of metastasis in TNBC, acting by elevating the proliferation and invasive properties of the tumor cells, and their resistance to chemotherapy (19, 20, 23, 24, 26, 97–99). Moreover, MSC-derived CCL2 has attracted macrophages to TNBC tumors, activating them to secrete CXCL8, thus leading to an overall increase in tumor-associated macrophages and endothelial cells (21).

The above studies strengthen the relevance and importance of our observations on the strong induction of CXCL8, CCL2 and CCL5 when TNBC cells interacted with MSCs/CAFs in the context of pro-inflammatory stimuli, introduced by TNFα and IL-1β. As noted above, both TNFα and IL-1β were found to be responsible for increased aggressiveness in TNBC, and in several studies were connected to increased pro-malignancy functions of MSCs/CAFs. For example, the findings by Shi and colleagues indicated that TNFα-activated MSCs promoted *via* CXCR2 and CCR2 ligands the metastatic ability of murine TNBC cells (63, 65). TNFα-primed MSCs were also found to reprogram neutrophils to acquire immunosuppressive functions (64). Other studies demonstrated that MDA-MB-231-derived CM elevated IL-1β release by MSCs, increasing their pro-inflammatory nature (100). In parallel, MSC-derived IL-1β increased the proliferation and chemoresistance of MDA-MB-231 TNBC cells (60).

However, these studies did not address the wider scope of the tumor-stroma-inflammation network, and did not identify the roles of pro-inflammatory cytokines such as TNFα and IL-1β in regulating TNBC-stroma interactions. Here, our current study provides novel findings, emphasizing the need for both TNBC:MSC cross-talk and pro-inflammatory signals delivered by TNFα and IL-1β, in order to achieve the most substantial levels of pro-metastatic activities: high levels of pro-metastatic chemokines, CXCL8, CCL2 and CCL5, angiogenesis, and tumor cell migration and invasion. Moreover, our findings suggest that previous studies on TNFα-treated MSCs that induced anti-tumor activities in TNBC tumors (101–104) may have overlooked the actual setting that takes place *in vivo*, when TNBC cells interact with MSCs in the presence of TNFα stimulation.

Of major importance in this context is the fact that CXCL8 was revealed in our current study as a key player in mediating the pro-metastatic functional effects of the inflammation-driven tumor-stroma networks in TNBC: angiogenesis, migration-related morphology of the tumor cells, as well as cancer cell migration and invasion. The effects of CXCL8 down-regulation on these pro-metastatic functions in TNBC was pronounced, and our results suggest that it can probably act in cooperativity with other factors that are produced under these network conditions to promote the aggressiveness of TNBC cells that interacted with stromal cells in the context of the pro-inflammatory TME.

Here, it is interesting to note that the tumor-stroma-inflammation networks established by luminal-A cells were less potent or differently active than those generated in TNBC, in all aspects: chemokine production, angiogenesis, and tumor cell morphology, migration and invasion. These findings may reflect the fact that TNBC cells and luminal breast tumor cells interact differently with fibroblasts (105). They also agree well with our TCGA results demonstrating lower expression levels of TNFα, IL-1β and of the three chemokines in luminal-A patients compared to basal patients. Ultimately, these findings may provide a partial explanation to the more aggressive clinical course of TNBC tumors compared to luminal-A tumors.

Overall, our observations suggest that at the TME of TNBC tumors, which is enriched with TNFα and IL-1β, the two pro-inflammatory cytokines regulate tumor-stroma interactions that occur at the tumor site, and that under these conditions the *in vivo* aggressiveness of the tumor cells is increased. It would be interesting to establish similar systems with murine TNBC cells and investigate the possible effects of similar tumor-stroma-inflammation networks and of specific corresponding mouse chemokines on the immune and inflammatory contextures of mice tumors and metastases. Such systems may also enable further analyses that correlate the extent of stroma cell presence with the extent of expression of pro-inflammatory cytokines and chemokines, as well as with patterns of tumor cell migration and angiogenesis.

The tumor-stroma-inflammation network identified in our study suggests that inhibiting the activities of TNBC-typical pro-inflammatory cytokines, such as TNFα and IL-1β would halt tumor-stroma interactions that stand in the basis of TNBC progression. Indeed, inhibitors of TNFα and IL-1β are in clinical use in inflammatory diseases and were found to inhibit the aggressiveness phenotype of TNBC cells (54, 57). Obviously, implementation of inhibitory modalities to these cytokines in the *in vivo* and even more so in the clinical setting would require improved understanding of the entire context of their activities; for example, the activation and regulatory networks of TNFα and its TNFR1 and TNFR2 receptors take place at multiple regulatory levels that need improved understanding.

These considerations emphasize the relevance of the metastatic chemokines that are elevated due to the activity of the tumor-stroma-inflammation triage, particularly CXCL8, to therapy. Inhibitors of the axes of CXCL8, CCL2 and CCL5 and their receptors are also available (20, 106, 107), suggesting that treatments of TNBC cancers with combination therapies of chemokines and pro-inflammatory cytokines may provide novel treatment options for TNBC patients.

## Ethics Statement

All procedures involving experimental animals were approved by Tel Aviv University Ethics Committee, and were performed in compliance with local animal welfare laws, guidelines, and policies.

## Author Contributions

YL generated all data, and was extensively engaged in study design and manuscript preparation. SL was involved in setting up the research systems. TM contributed to qRT-PCR studies. LR-A and DM participated in ELISA studies of luminal-A and TNBC cell lines. SW contributed to conception of research at its initial stages and CK participated in TGCA analyses. AB-B was the principal investigator, responsible for the entire study at all stages (conception, design and data accumulation), as well as manuscript preparation.

### Conflict of Interest Statement

The authors declare that the research was conducted in the absence of any commercial or financial relationships that could be construed as a potential conflict of interest.
