# Supplementary material for: Tumor-Stroma-Inflammation Networks Promote Pro-metastatic Chemokines and Aggressiveness Characteristics in Triple-Negative Breast Cancer
Source: Front Immunol. 2019 Apr 12;10:757. doi: 10.3389/fimmu.2019.00757 (PMC6473166; doi:10.3389/fimmu.2019.00757)
Supplement: Supplementary file 1 [file Data_Sheet_1.PDF]

Supplementary Figure 1

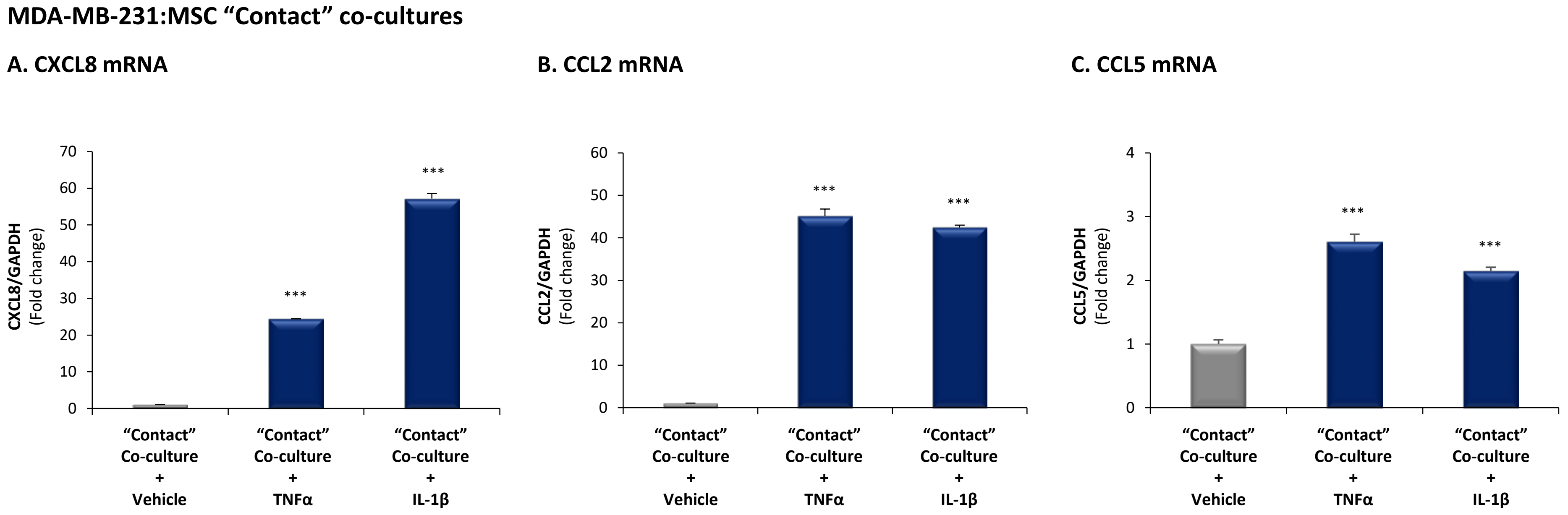

Supplementary Figure 1

In response to TNFα and IL-1β stimulation, CXCL8, CCL2 and CCL5 are up-regulated at the mRNA level in MDA-MB-231:MSC "Contact" co-cultures

MDA-MB-231:MSC "Contact" co-cultures were stimulated by TNFα (10 ng/ml), IL-1β (350 pg/ml) or vehicle for 7 hrs. The expression levels of CXCL8, CCL2 and CCL5 were determined by qRT-PCR. ***p<0.001 for differences between cytokine-stimulated and vehicle-treated co-cultures. The results are of a representative experiment of n=3 independent experiments, performed with MSCs of 2 different donors.

Supplementary Figure 2

CXCL8 up-regulation

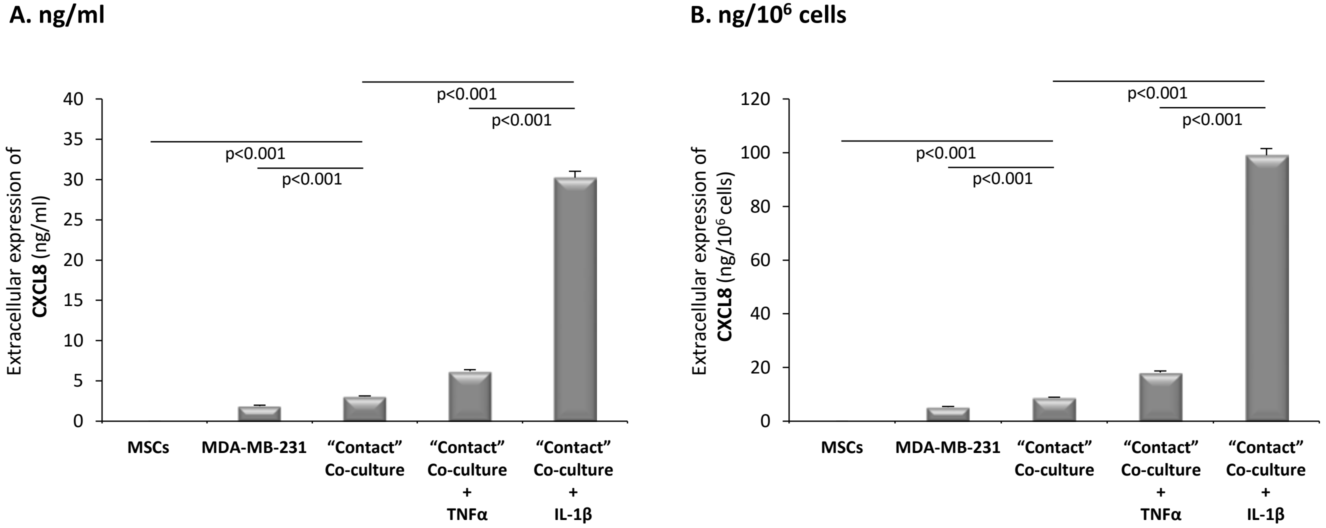

Supplementary Figure 2

Elevated CXCL8 levels, induced by TNFα- and IL-1β-stimulation of MDA-MB-231:MSC "Contact" co-cultures, do not result from elevated proliferation of the cells
MDA-MB-231 cells and MSCs were grown either alone or in "Contact" co-cultures and were then stimulated by TNFα (10 ng/ml), IL-1β (350 pg/ml) or vehicle for 7 hrs, as described in Figure 3. At the end of the experiment, the cells were counted and in parallel the extracellular expression of CXCL8 in cell supernatants was determined by ELISA. Data are presented as **(A)** CXCL8 levels in values of ng/ml and **(B)** CXCL8 levels in values of ng/10^6 cells. n=1; technical standard deviations of each sample are demonstrated.

Supplementary Figure 3

MDA-MB-231:MSCs – HPMEC sprouting in response to CM

A. Quantification

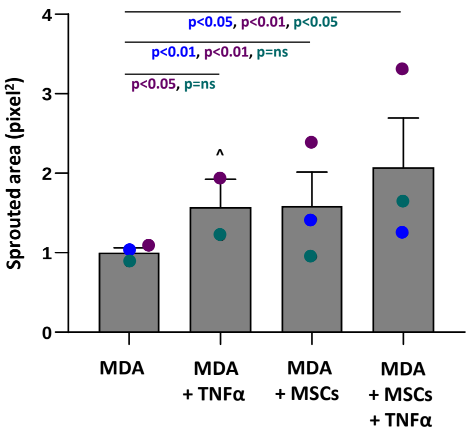

B. Representative images

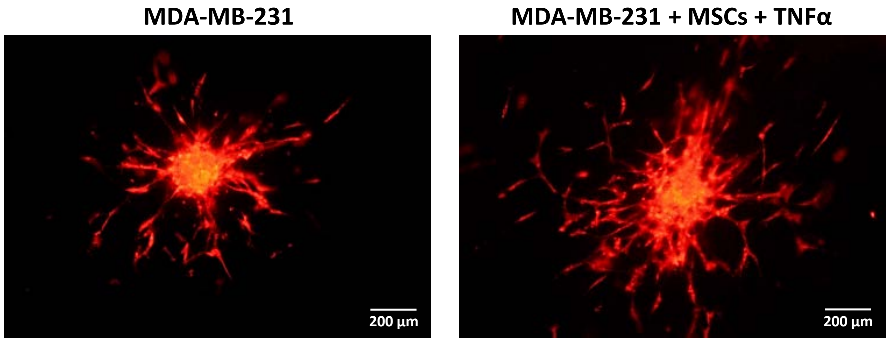

Supplementary Figure 3

The tumor-stroma-inflammation network in TNBC promotes endothelial cell sprouting

Studies of HPMEC sprouting out of 3D spheroids, in response to TNFα-free CM derived from TNFα-stimulated “Contact” co-cultures of MDA-MB-231 cells (“MDA”) and MSCs (TNFα: 10 ng/ml) or in response to TNFα-free CM of tumor cells alone (treated by vehicle), of tumor cells treated by TNFα (10 ng/ml) or of tumor cells co-cultured with MSCs. **(A)** Averages and standard errors of sprout quantification (in multiple photos in each experiment) in n=3, by ImageJ (^ In this groups, n=2). The Figure provides p values of each of the three experimental repeats, comparing sprouting when HPMEC were treated with TNFα-free CM of the different conditions *vs.* CM of MDA-MB-231 cells alone. **(B)** Representative photos of HPMEC sprouting in the two groups that were significantly different in each of the three experimental repeats : HPMEC treated with TNFα-free CM derived from “Contact” co-cultures of MDA-MB-231 cells *vs.* CM derived from MDA-MB-231 cells. Photos and their quantifications were derived from studies performed with MSCs of 2 different donors. Bar, 200 µm.

Supplementary Figure 4

Representative photos of migrating tumor cells

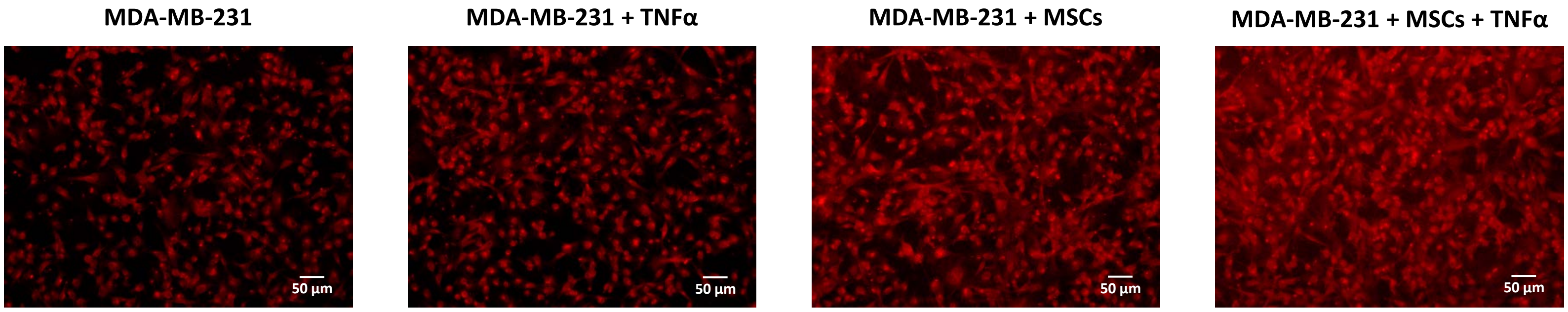

Supplementary Figure 4
TNBC cells acquire elevated migratory capacity upon co-culturing with MSCs in the presence of TNFα
The Figure demonstrates representative photos of migrating tumor cells, whose quantitative analysis was provided in Figure 9A2, in which the migration of mCherry-MDA-MB-231 cells was determined under different conditions. Tumor cells were identified by Abs to RFP/mCherry, followed by DyLight 550-conjugated secondary Abs (preliminary studies using DyLight550 signals and Hemacolor staining further indicated that ~100% of migrating cells were the tumor cells). Photos are representatives of many fields photographed in a representative experiment of n=3 independent experiments, performed with MSCs of 3 different donors. Bar, 50 µm.

Supplementary Figure 5

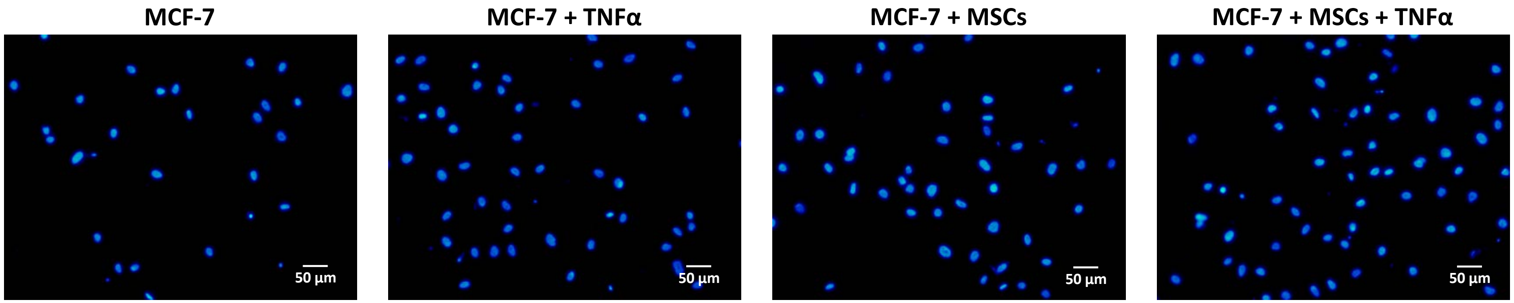

Supplementary Figure 5
Migration of MCF-7 cells upon co-culturing with MSCs in the presence of TNFα
This Figure is complementary to Figure 9B2, and it demonstrates representative photos of migrating cells in co-cultures of Hoechst-MCF-7 cells and MSCs, stimulated by TNFα (10 ng/ml) (Bar, 50 µm). Photos are representatives of many fields photographed in a representative experiment of n>3 independent experiments, performed with MSCs of 3 different donors.

Supplementary Table 1

Gene	Forward primer (5'-3')	Reverse primer (5'-3')
CXCL8	TTCTGCAGCTCTGTGTGAAG	CAGTGTGGTCCACTCTCAAT
CCL2	AGTCTCTGCCGCCCTTCT	GTGACTGGGGCATTGATTG
CCL5	ACCCTGCTGCTTTGCCTACA	ACACACTTGGCGGTTCTTTC
GAPDH	CCACATCGCTCAGACACCAT	CAACAATATCCACTTTACCAGAGTTAA

Supplementary Table 1
Primers used in qRT-PCR analyses. For detailed description of qRT-PCR procedures, please see “Materials and methods”.
